# Targeting LAG-3, TIM-3, and TIGIT for cancer immunotherapy

**DOI:** 10.1186/s13045-023-01499-1

**Published:** 2023-09-05

**Authors:** Letong Cai, Yuchen Li, Jiaxiong Tan, Ling Xu, Yangqiu Li

**Affiliations:** 1 Key Laboratory for Regenerative Medicine of Ministry of Education, Institute of Hematology, School of Medicine, Jinan University, Guangzhou, 510632 China; 2 Key Laboratory of Viral Pathogenesis & Infection Prevention and Control (Jinan University), Ministry of Education, Guangzhou, 510632 China

**Keywords:** LAG-3, TIM-3, TIGIT, Solid tumor, Leukemia

## Abstract

In one decade, immunotherapy based on immune checkpoint blockades (ICBs) has become a new pillar of cancer treatment following surgery, radiation, chemotherapy, and targeted therapies. However, not all cancer patients benefit from single or combination therapy with anti-CTLA-4 and anti-PD-1/PD-L1 monoclonal antibodies. Thus, an increasing number of immune checkpoint proteins (ICPs) have been screened and their effectiveness evaluated in preclinical and clinical trials. Lymphocyte activation gene-3 (LAG-3), T cell immunoglobulin and mucin-domain-containing-3 (TIM-3), and T cell immunoreceptor with immunoglobulin and tyrosine-based inhibitory motif (ITIM) domain (TIGIT) constitute the second wave of immunotherapy targets that show great promise for use in the treatment of solid tumors and leukemia. To promote the research and clinical application of ICBs directed at these targets, we summarize their discovery, immunotherapy mechanism, preclinical efficiency, and clinical trial results in this review.

## Introduction

Immune suppression resulting from cancer cell immune escape is closely related to tumor development and progression, treatment resistance, and poor prognosis. The mechanisms underlying immune suppression include complex steps and factors, and a key aspect is cytotoxic immune cell (CD8^+^ T cells and natural killer (NK) cell) exhaustion. T/NK cell exhaustion is generally recognized by the increased expression of several immune checkpoint proteins (ICPs), such as programmed cell death protein 1 (PD-1), cytotoxic T lymphocyte antigen-4 (CTLA-4), lymphocyte activation gene-3 (LAG-3), T cell immunoglobulin and mucin-domain-containing-3 (TIM-3), T cell immunoreceptor with immunoglobulin and ITIM domain (TIGIT), and B and T lymphocyte attenuator. These ICPs then inhibit the tumor-killing capacity of T/NK cells by ligating with respective ligands expressed on antigen-presenting cells (APCs), tumor cells, and other cells in the tumor microenvironment (TME) [1–3]. These ICPs have been aptly called the “brakes” of T and NK cells. The preponderance of evidence has shown that blocking the binding of ICPs or their ligands reverses the antitumor immune response of immune cells, resulting in tumor regression [4, 5]. In addition to expression on immune cells, some ICPs are expressed in tumor cells, sometimes promoting the proliferation and survival of these cells. Thus, blocking ICPs may have a “one stone, two birds” effect in tumor treatment [6]. Since the approval of the first immune checkpoint inhibitor (ICI), ipilimumab (an anti-CTLA-4 monoclonal antibody (mAb)), for the treatment of unresectable and metastatic melanoma by the US Food and Drug Administration (FDA) in 2014, immunotherapy based on immune checkpoint blockers (ICBs) has been approved for the treatment of more tumor types in earlier disease stages. Currently, four types of ICIs (anti-PD-1, PD-L1, CTLA-4, and LAG-3 mAbs) have been approved by the FDA for tumor treatment [7, 8]. Other ICIs, such as TIM-3 and TIGIT inhibitors, have been extensively evaluated in clinical trials as treatments for different solid tumors and leukemia [9, 10].

Due to varying expression and coexpression levels of other ICPs in different tumors, the efficiency of ICBs varies greatly. For example, while PD-1/PD-L1 blockade monotherapy has achieved a satisfactory response in patients with different cancers, a meta-analysis demonstrated that approximately four-fifths of patients do not respond to PD-1/PD-L1 monotherapy in clinical trials [11]. The reasons for resistance to PD-1/PD-L1 blockade remain unclear, but related factors may include the lack of PD-L1 expression [11]; heterogeneity in the TME, including reduced immune cell diversity [12–14]; the lack of active immune cells [15]; the existence of specific TCR clones; and the coexpression of other ICPs [16–18]. Thus, numerous clinical trials are currently evaluating the efficiency of different ICI monotherapies or combined therapies in different tumors. PD-1/PD-L1 and CTLA-4 blockers are widely used and extensively studied in the clinic, but the characteristics and efficiency of LAG-3, TIM-3, and TIGIT blockers have not been completely described. In this review, we describe the gene and protein characteristics, biological functions, and abnormal expression profiles of LAG-3, TIM-3, and TIGIT in solid tumors and leukemia. Most importantly, we summarize the advancements shown by studies related to the development of three ICPs from the bench to bedside and discuss their advantages and limitations.

## Structural characteristics and biological functions of LAG-3, TIM-3, and TIGIT

**Fig. 1:**
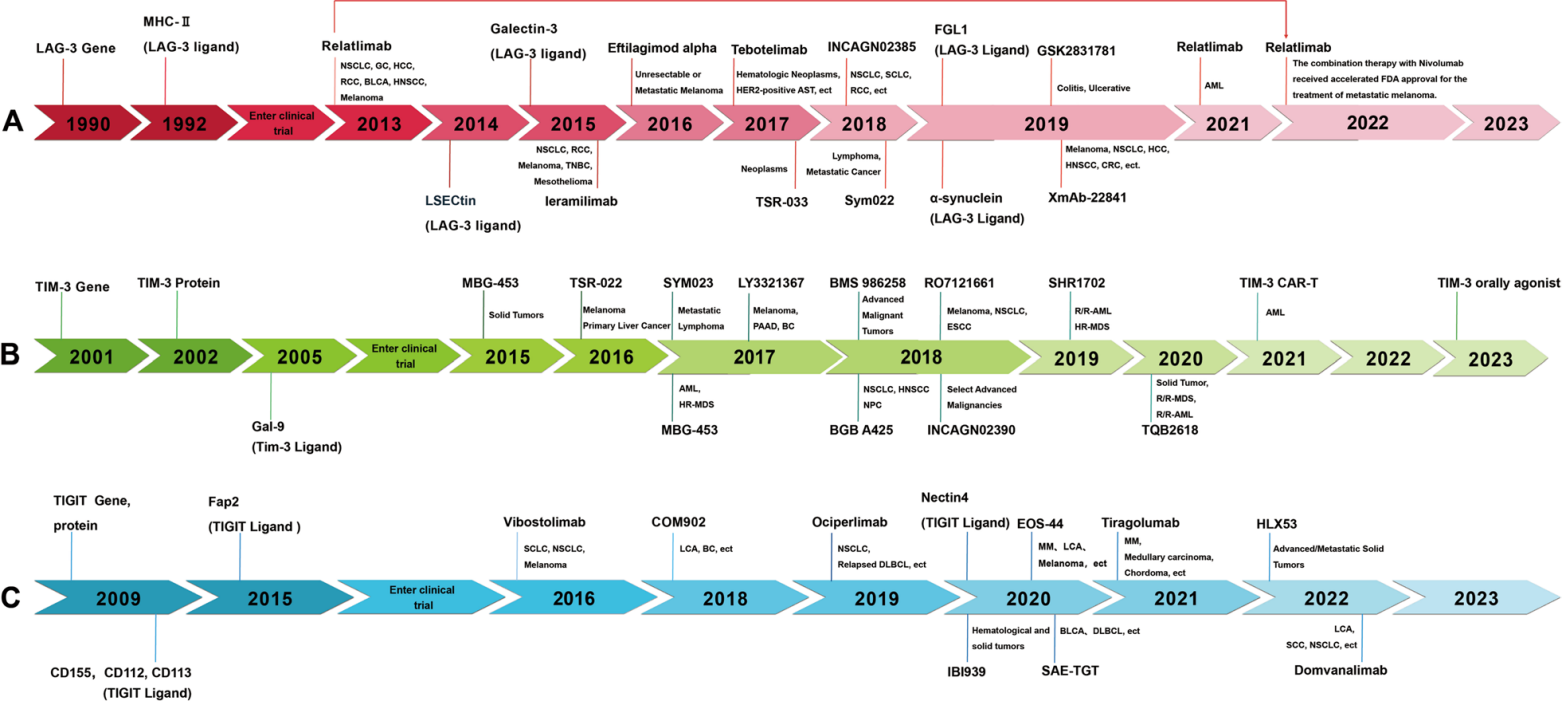
Timeline of discovery and antibody development of LAG-3, TIM-3 and TIGIT. A. LAG-3, B. TIM-3, C. TIGIT. *Notes*: AML: Acute Myelocytic Leukemia; AST: Advanced Solid Tumors; BC: Breast Cancer; BLCA: Bladder Cancer; BLCA: Bladder Cancer; CAR-T: Chimeric Antigen Receptor T Cell Immunotherapy; CRC: Colorectal Cancer; DLBCL: Diffuse Large B cell Lymphoma; ESCC: Esophageal Squamous Cell Carcinoma; Fap2: Fibroblast activation protein 2; FGL-1: Fibrinogen-like protein 1; Gal-9: Galectin-9; GC: GC; HCC: Hepatocellular Carcinoma; HNSCC: Head and neck squamous cell carcinoma; LCA: Lung cancer; LSECtin: Liver and lymph node sinusoidal endothelial cell C-type lectin; MDS: Myelodysplastic Syndromes; MHC-II: MHC class II; MM: Multiple Myeloma; NPC: Nasopharyngeal Carcinoma; NSCLC: Non-small Cell Lung Cancer; OV: Ovarian Cancer; PAAD: Pancreatic Cancer; RCC: Renal Cell Carcinoma; SCC: Squamous Cell Carcinoma; SCLC: Small Cell Lung Carcinoma; TNBC: Triple-Negative Breast Cancer

LAG-3, TIM-3, and TIGIT were identified in 1990, 2001, and 2009, respectively. The structures of these three ICPs are distinct, but their biological functions, namely inducing the exhaustion of immune cells and mediating immune suppression, are similar. The timeline of the discovery and antibody development for LAG-3, TIM-3, and TIGIT is shown in Fig. 1. The details of LAG-3, TIM-3, and TIGIT ligands are listed in Table 1.

**Table 1:** The ligands for LAG-3, TIM-3, TIGIT and their interactions

IC	Ligands	Expression	Mechanism of action	Ref.
LAG-3	MHC-II	B cells, MON-Mø, DCs, some activated T cells	Negatively regulates T cell responses	[24]
	FGL-1	FGL-1 protein is primarily secreted from hepatocytes	Inhibiting antitumor immune responses	[28]
	α-synuclein	Neurons, heart, muscle, and other tissues	LAG-3 can recognize α-synuclein fibrils and affect its endocytosis and intercellular transmission, contributing to PD	[223]
	Gal-3	Tumor cells, macrophages, epithelial cells, fibroblasts, activated T cells	Inhibiting antitumor T cell responses	[27]
Tim-3	LSECtin	Liver, tumor-associated macrophages, and other tumor tissues	Inhibiting antitumor T cell responses	[26]
	Gal-9	APC, MDSCs, Naive CD4 T cells, plasma	Gal-9 mainly induces calcium to flow into the intracellular area of Th1 cells and induces apoptosis	[224]
	PtdSer	Released from apoptotic cells	PtdSer and TIM-3 binding contributes to the clearance of apoptotic bodies and antigen cross-presentation by Tim-3^+^ DCs	[104]
	CEACAM-1	DCs, monocytes, macrophages, and activated T cells	CEACAM-1/TIM-3 complex formation has a crucial role in regulating autoimmunity and antitumor immunity	[102]
	HMGB1	Proliferating tissues or estrogen stimulated cancer cells	Blocking activation and suppresses innate immune responses to nucleic acids	[103]
TIGIT	CD155	DCs, T cells, B cells, macrophages	Increasing the IL-10 secretion	[51]
	CD113	Liver, testes, lungs, placenta, and kidneys	Inhibition of T cell and NK cell activity	[121]
	CD112	Hematopoietic and non-hematopoietic tissues	Inhibiting the activation of T cells and NK cells	[225]
	Nectin4	Tumor cells	Inhibiting NK cell activity	[55]
	Fap2	Tumor cells	Inhibiting NK cell toxicity and T cell activity	[56]

*APCs* antigen presenting cell; *CEACAM-1* carcinoembryonic antigen-related cell adhesion molecule 1; *DCs* dendritic cells; *Fap2* fibroblast activation protein 2; *FGL-1* fibrinogen-like protein 1; Gal-3: Galectin-3; Gal-9: Galectin-9; HMGB1: High Mobility Group Box 1; LSECtin: Liver and lymph node sinusoidal endothelial cell C-type lectin; MDSCs: Myeloid-derived suppressor cells; MHC-II: Major histocompatibility complex class II; MON-Mø: Monocytes–macrophages; Nectin4: Nectin cell adhesion molecule 4; NK: Natural killer cell; PD: Parkinson’s disease; and PtdSer: Phosphatidylserine

### LAG-3

LAG-3 (also named CD223 or FDC) was identified in 1990 by Triebel and colleagues while screening molecules that were selectively expressed in F5 cells, a CD3-negative interleukin (IL)-2-dependent NK cell line [19]. *LAG-3* is located on the distal part of the short arm of chromosome 12 (12p13.31) in humans and chromosome 6 (6;6 F2) in mice. *LAG-3* encodes a 525 amino acid protein that carries a signal peptide of 23 amino acids and an approximately 70 kDa mature Type I transmembrane glycoprotein in the immunoglobulin superfamily. To date, three isomers of* LAG-3* have been identified; they range from LAG-3 protein isoform 1 precursor to LAG-3 protein isoform 3 precursor [20]. The structure of LAG-3 is different from that of CD3 and CD8, but it is highly homologous to that of CD4; LAG-3 consists of a transmembrane region, an extracellular region, and a cytoplasmic region. The extracellular structure consists of four IgSF domains, namely D1, D2, D3, and D4, which are critical for binding ligands. The D1 domain contains a loop domain rich in proline and an in-chain disulfide bond, which is species-specific and is in the V immunoglobulin superfamily. However, D2, D3, and D4 belong to the C2 family. The cytoplasmic region of LAG-3 consists of three parts: a serine phosphorylation site S454; a highly conserved “KIEELE” motif, which is known to be highly conserved in primates, mice, and rats; and a glutamate-proline dipeptide repeat motif (EP sequence). Soluble LAG-3 (sLAG-3) detaches from the cell membrane through the action of the metalloprotein (ADAM10/17) enriched in lipid rafts at the 20-aa connecting peptide between D4 and the transmembrane domain [20, 21].

At present, the LAG-3-related signal remains unclear. Louzalen et al. identified a novel protein called LAG-3-related protein (LAP) that binds to repetitive EP sequences in the LAG-3 intracellular region and may be involved in the downregulation of the CD3/T cell receptor (TCR) activation pathway (19). In addition, LAP may facilitate LAG-3 colocalization with CD3, CD4, and/or CD8 within glycosphingolipid-enriched microdomains (lipid rafts) to form immune synapses that regulate TCR signaling [22]. Studies have also shown that the KIEELE motif in the cytoplasmic domain is crucial for the activity of LAG-3. A single lysine residue (K468) in the conserved “KIEELE” sequence may recruit or mediate the activation of currently unknown signaling molecules, leading to downstream protein signaling [23].

LAG-3 is mainly expressed on activated T and B cells, NK cells, and dendritic cells (DCs) under physiological conditions, and it can negatively regulate T cell function [24]. Interestingly, LAG-3 was also found to be expressed on a proportion of malignant B cells from patients with diffuse large B cell lymphoma (DLBCL) [25]. To date, five LAG-3 ligands have been identified: MHC class II (MHC-II) [24], liver sinusoidal endothelial cell lectin (LSECtin) [26], galectin-3 [27], α-synuclein fibrils, and fibrinogen-like protein 1 (FGL-1) [28, 29].

LAG-3 can negatively regulate the function of T cells, exerting important effects on maintaining the homeostasis of the immune system under normal physiological conditions and promoting tumor cell immune escape in the TME [30]. LAG-3 also mediates bidirectional signaling in APCs. During Treg-DC interactions, engagement of LAG-3 on Tregs can enhance Treg activity to promote immune tolerance and indirectly inhibit DC function. Given its important biological role, LAG-3 is considered a promising target for cancer immunotherapy [31]. To date, more than twenty anti-LAG-3 antibodies have been used in clinical trials for cancer immunotherapy. Relatlimab, the first commercially developed anti-LAG-3 mAb, was entered into clinical trials in 2013, and it received FDA approval in March 2022, along with the PD-1 inhibitor nivolumab in the combination treatment Opdualag (Bristol Myers Squibb), which is used for the treatment of unresectable or metastatic melanoma.

### TIM-3

TIM-3 is a member of the* TIM* gene family, and it was identified in 2001 during a study of asthma susceptibility genes in congenic inbred mice. Murine TIM-3 is located on chromosome 11 within the Tapr region, and the human *TIM-3* gene is located on chromosome 5 at q33.2. The full-length human *TIM-3* cDNA is 906 bp and encodes a predicted membrane protein of 281 amino acid residues [32]. *TIM-3* isoforms were identified in 2003, and an 800-bp amplicon was shown to encode an alternatively spliced soluble form of TIM-3 (sTIM-3) [33]. Initially, it was hypothesized that sTIM-3 competitively prevents TIM-3 from binding the TIM-3 ligand, which results in Th1 cells continuing to proliferate and perform effector functions [34]. In contrast, however, other studies suggested that sTIM-3 binds to ligands on T cells and suppresses antitumor immunity [33, 35]. TIM-3 is a single transmembrane molecule with an extracellular tail that carries an N-terminal immunoglobulin variable domain. This domain is successively followed by a mucin domain with glycosylation sites, a peptide linker with N-linked glycosylation sites, a transmembrane domain, and the C-terminus [36, 37]. TIM-3 contains a conserved region with five tyrosine residues. Two residues, Y265 and Y272 in humans, are assumed to be phosphorylated after the interaction of TIM-3 with its ligands. Itk, a Tec family tyrosine kinase, and Fyn and Lck, two kinases in the Src family, are involved in the TIM-3 signaling pathway. The activation of these tyrosine kinases leads to the accumulation of proteins with SH2 domains, such as the p85 subunit of phosphoinositide 3-kinase and phospholipase C-γ1, in the cytoplasmic tail of TIM-3. Furthermore, TIM-3 activation enhances nuclear factor of activated T cells and nuclear factor kB (NF-κB) activity through its interaction with zeta-chain-associated protein kinase 70 and SLP-76, which are components of the TCR signaling pathway. Importantly, the SH2 domain-binding motif is a trans-regulatory sequence that controls TIM-3-mediated signal transduction [38]. In addition, human leukocyte antigen-B-associated transcript 3 directly binds to the cytoplasmic tail of TIM-3 and prevents signaling in the absence of TIM-3 ligand(s) [38].

To date, TIM-3 has been found to be expressed on T cells (except for Th2 cells), and other immune cells, such as NK cells, macrophages, DCs, myeloid-derived suppressor cells, and mast cells. Moreover, TIM-3 is also expressed on certain malignant cells, such as melanoma [39, 40], myeloid leukemia [41], non-small cell lung cancer (NSCLC) [42], prostate cancer [43], osteosarcoma [44], colon carcinoma [45], and hepatocellular carcinoma (HCC) cells [46]. When TIM-3 binds to a ligand, immune cell or adaptive immune cell maturation and activation is attenuated, which is beneficial to tumor cell proliferation and survival. To date, four TIM-3 ligands have been identified. The first and most extensively characterized ligand is galectin-9 (Gal-9), followed by high-mobility group protein B1 (HMGB1), phosphatidylserine (PtdSer), and carcinoembryonic antigen cell adhesion molecule 1 (CEACAM-1) (Table 1). Different patterns of TIM-3 and ligand binding in various types of cells may result in different biological effects; e.g., HMGB1 binds to TIM-3 in different contexts that does not always lead to the same outcome [47]. In addition to the four aforementioned ligands, retinoic acid-inducible gene I (RIG-I), which is a member of the (RIG-I)-like receptor family, has also been reported to interact directly with Tim-3. Specifically, Tim-3 inhibits RIG-I expression in macrophages through the action of STAT1, promotes RIG-I ubiquitination and degradation through the action of the E3 ligase RNF-122, and subsequently inhibits type I interferon (IFN) production and antiviral activity [48–50]. The first anti-TIM-3 mAb, sabatolimab, was developed for use in solid tumor therapy, and it works by blocking the binding of TIM-3 to its ligands Gal-9 and PtdSer. To date, more than 33 TIM-3 mAbs have been evaluated in clinical trials as cancer immunotherapies.

### TIGIT

TIGIT was identified in 2009 through the combination of two genome-wide search strategies used in studies to determine whether activated human T cells express costimulatory or inhibitory molecules, particularly genes expressed in T cells and NK cells [51]. The mouse *TIGIT *gene is located at the B4 position of chromosome 16, while the human *TIGIT* gene is located at q13.31 on chromosome 3. Human *TIGIT* cDNA is 2926 bp in length and encodes 244 amino acids. Six variants encode *TIGIT* isoforms [51, 52]. TIGIT is expressed on lymphocytes, including Tregs, memory T cell subsets, and NK cells [53], and its expression can be upregulated when these cells are activated [51]. In addition, TIGIT has been reported to be expressed on tumor cells in mice [54].

It is now believed that there are five TIGIT ligands, namely CD155 (also known as PVR), CD112, CD113, Nectin4 [55], and Fab2 [56]. TIGIT encodes a protein carrying an immunoglobulin variable domain, a transmembrane domain, and an immunoreceptor tyrosine-based inhibitory motif (ITIM). Human TIGIT shares 58% sequence identity with mouse TIGIT, and the ITIM-containing sequence is identical in the cytoplasmic tails of mouse and human TIGIT [51, 52]. The binding of TIGIT to its ligands triggers the activation of a series of signaling pathways that affect the function of immune cells and the immune response, thereby causing an overall immune suppressive response in cells. Notably, in addition to binding with ligands, TIGIT can carry out its the immune suppression function via interference of the co-stimulation signaling in T cells mediated by CD226 or CD96 [51, 57].

More than 45 types of TIGIT inhibitors have been developed, and most of them are used in clinical practice for solid tumors and leukemia; however, only a few anti-TIGIT mAbs have been entered into Phase III clinical trials. IBI939 is the first anti-TIGIT mAb approved for clinical use in China, and it is currently in Phase I trials for patients with leukemia and solid tumors [58]. Another mAb, tiragolumab, is a fully human anti-TIGIT IgG1/kappa mAb developed by Roche that carries a complete Fc region that blocks the binding of TIGIT to its receptor CD155 [59].

## Upregulating the expression of LAG-3, TIM-3 and TIGIT in solid tumors and leukemia

In solid tumors and leukemia, ICPs are generally increased expression on immune cells and bind to ligands on malignant cells or APCs, leading to the depletion of T/NK cells or the disruption of their antitumor function [39]. ICPs can also be expressed on malignant cells and may promote their proliferation [60–62]. For example, TIM-3 is expressed in patients with solid tumors and leukemia [42, 62, 63]. The expression of LAG-3, TIM-3 and TIGIT in solid tumors and leukemia and their correlation with clinical outcomes are summarized in Table 2.

**Table 2:** LAG-3, TIM-3, and TIGIT expression and their clinical significance in solid tumors and leukemia

IC	Expression on immune cells	Expression on tumor cells	disease	Association with Clinical outcome	Ref.
LAG-3	Activated CD4^+^ and CD8^+^ T cells	–	Melanoma	Impairs immune cells function and antitumor immune response	[76, 78]
	TILs	–	NSCLC	Associate with a worse prognosis	[3]
	TILs	–	HNSCC	High pathological grade, larger tumor size and positive lymph node status	[4, 6]
	Peripheral CD4^+^ and CD8^+^ T cells, TILs	–	STS	High pathological grades, advanced tumor stage, and poor prognosis	[7]
	CD8^+^ TIL, DC	–	RCC	Associated with a poorer prognosis for RCC in humans	[78]
	TILs, tumor-associated perivascular lymphocytes	–	Glioblastoma	Correlated with significantly less IFN-γ release upon activation and is a marker of T cell exhaustion	[20, 61]
	TILs	–	HCC	Positively associated with more types of cirrhosis and advanced cancer	[71, 226]
	TILs	–	PAAD	Significantly reduce disease-free survival in patients	[25, 26]
	Leukemia-tolerant CD8^+^ T cells	Leukemia cells	HMs	Promoting T cell dysfunction	[4]
	CD4^+^/CD8^+^ T cells	–	GC	Improve the prognosis of patients with advanced gastric cancer who receive anti-programmed death-1 antibody therapy	[67]
	TILs at the tumor front	–	Stage II CRCA	Predict better treatment outcomes in both the entire stage II and the subgroup of stage II microsatellite-stable tumors	[84]
	TILs	–	TNBC, Her2 + BC	The infiltration of LAG-3 lymphocytes ameliorates OS in TNBC and Her2 + BC	[7]
	CD4^+^ Tregs, CD8^+^ T cells, TAMss	Malignant B cell	DLBCL	Associated with poor survival and poor prognosis	[25]
TIM-3	Mast cells, antigen-specific CD8^+^ T cells, NK cells	Melanoma cells	Melanomas	Promote tumor progression	[39, 60, 61]
	TADCs, TILs	Tumor cells	Lung cancer	Lower survival	[47] [42]
	TADCs, CD8^+^TILs	MC38 cells, colon cancer cells, tumor tissues	CC	Poor prognosis and Inhibition of tumor progression	[45, 152]
	TILs, TAMs, APC	HCC, HBV-associated HCC	HCC	Lower survival, paralleled the grades of HCC, Immunotherapy resistance	[93, 138, 227]
	CD8^+^ TILs	Lymphoma endothelial cells	NHL	Promote tumor progression	[63, 74]
	CD4^+^, CD8^+^T cells	LSCs	AML, MDS	Poor prognosis and relapse after allo-HSCT	[98, 228, 229]
TIGIT	CD8^+^T cells	–	AML	TIGIT expression on CD8^+^ T cells is elevated in AML patients and high-TIGIT correlates with primary refractory disease and leukemia relapse post-allo-SCT	[111]
	CD8^+^T cells	–	GC	Promote the development of advanced GC	[112]
	CD8^+^T cells	–	MM	Reduced tumor burden and improved survival	[230]
	CD8^+^TILs	CC	CRCA	Promote tumor growth	[54]
	CTLs	MCL	MCL	This led to a relapse after CAR-T cell therapy	[215]
	CD8^+^T cells	–	ESCC	Coexpression of TIGIT and PD-L1 leads to poor OS	[231]

Notes: AML: Acute Myelocytic Leukemia; BC: Breast Cancer; CAR-T: Chimeric Antigen Receptor T Cell Immunotherapy; CC: Colon Cancer, CTLs: Cytotoxicity T Lymphocytes, CRCA: Colorectal Cancer; DCs: Dendritic cells; DLBCL: Diffuse Large B cell Lymphoma; ESCC: Esophageal Squamous Cell Carcinoma; GC: Gastric Cancer; HBV: Hepatitis B; HCC: Hepatocellular Carcinoma; HNSCC: Head and Neck Squamous Cell Carcinoma; HSCT: Hematopoietic Stem Cell Transplantation; HMs: Hematological Malignancies; MCL: Mantle cell lymphoma; MDS: Myelodysplastic Syndromes; MM: Multiple myeloma; NHL: Non-Hodgkin lymphoma; NSCLC: Non-small Cell Lung Cancer; OS: Overall Survival; PAAD: Pancreatic cancer; RCC: Renal Cell Carcinoma; STS: Soft Tissue Sarcomas; TADCs: Tumor-associated Dendritic cells; TAMs: Tumor-associated Macrophages; TILs: Tumor-infiltrating Lymphocytes; TNBC: Triple-Negative Breast Cancer

### LAG-3

Overexpression of LAG-3 has been identified on tumor-infiltrating lymphocytes (TILs) in a number of solid tumors, including melanoma, glioma, NSCLC, head and neck squamous cell carcinoma (HNSCC), breast cancer (BC), gastric cancer (GC), and lymphoma, as well as in leukemia [64–75]. Early studies have found that in the melanoma context, metastatic lymph nodes were infiltrated not only by T cells (CD4^+^, CD8^+^, and Tregs) but also by a substantial percentage of NKT and NK cells that express LAG-3. LAG-3 expression mediated immune escape in melanoma cells by impairing immune cell function while also protecting against Fas- and drug-induced apoptosis via its interaction with MHC-II [76]. LAG-3 is expressed on a subset of human pDCs, and LAG-3^+^ pDCs are enriched in the tumor sites of melanoma patients. These LAG-3^+^ pDCs interact with MHC-II to induce Toll-like receptor-independent pDC activation, which may contribute to the formation of an immunosuppressive microenvironment by increasing IL-6 production [77, 78]. On the other hand, LAG-3 is expressed on a certain proportion of malignant B cells in patients with DLBCL and chronic lymphocytic leukemia (CLL), and digital protein spatial analysis showed that LAG-3 is strongly associated with macrophages in the TME. In contrast to TIM-3, no studies have reported the mechanism by which LAG-3 expressed on tumor cells promotes the proliferation of these cells [25].

Most data from different clinical investigations have indicated that higher LAG-3 expression is related to poor clinical outcomes. For example, increased LAG-3 expression is used to stratify patients with HNSCC into high-risk groups [79], and a high level of LAG-3 expression in soft tissue sarcoma tissues has been found to be significantly correlated with high pathological grade and late stage [69]. High LAG-3 expression levels have been associated with poorer prognosis for renal cell carcinoma (RCC) [80], poor overall survival (OS) of patients with either high- or low-grade glioma [81], and low disease-free survival in patients with pancreatic cancer [82]. In patients with NSCLC, positive LAG-3 expression has been associated with early postoperative relapse and worsened prognosis [72]. In addition, high expression of the *LAG-3* gene in patients with DLBCL is associated with poor survival and prognosis. Moreover, compared with LAG-3^low^/PD-L1^high^-expressing patients with DLBCL, those who expressed LAG-3^high^/PD-L1^high^ showed lower progression-free survival (PFS) and OS rates [25].

In contrast, an increase in the density of LAG-3 TILs indicated a trend toward higher OS times in patients with triple-negative BC and Her2-positive BC [83]. Interestingly, similar findings have been reported in which LAG-3 expression on TILs at the tumoral front predicted better treatment outcomes for all Stage II patients and for a subgroup of patients with Stage II microsatellite-stable colon cancer (CC) [84]. Whether these results are related to targeted therapy or immunotherapy is unclear, and further analysis is needed.

**Fig. 2:**
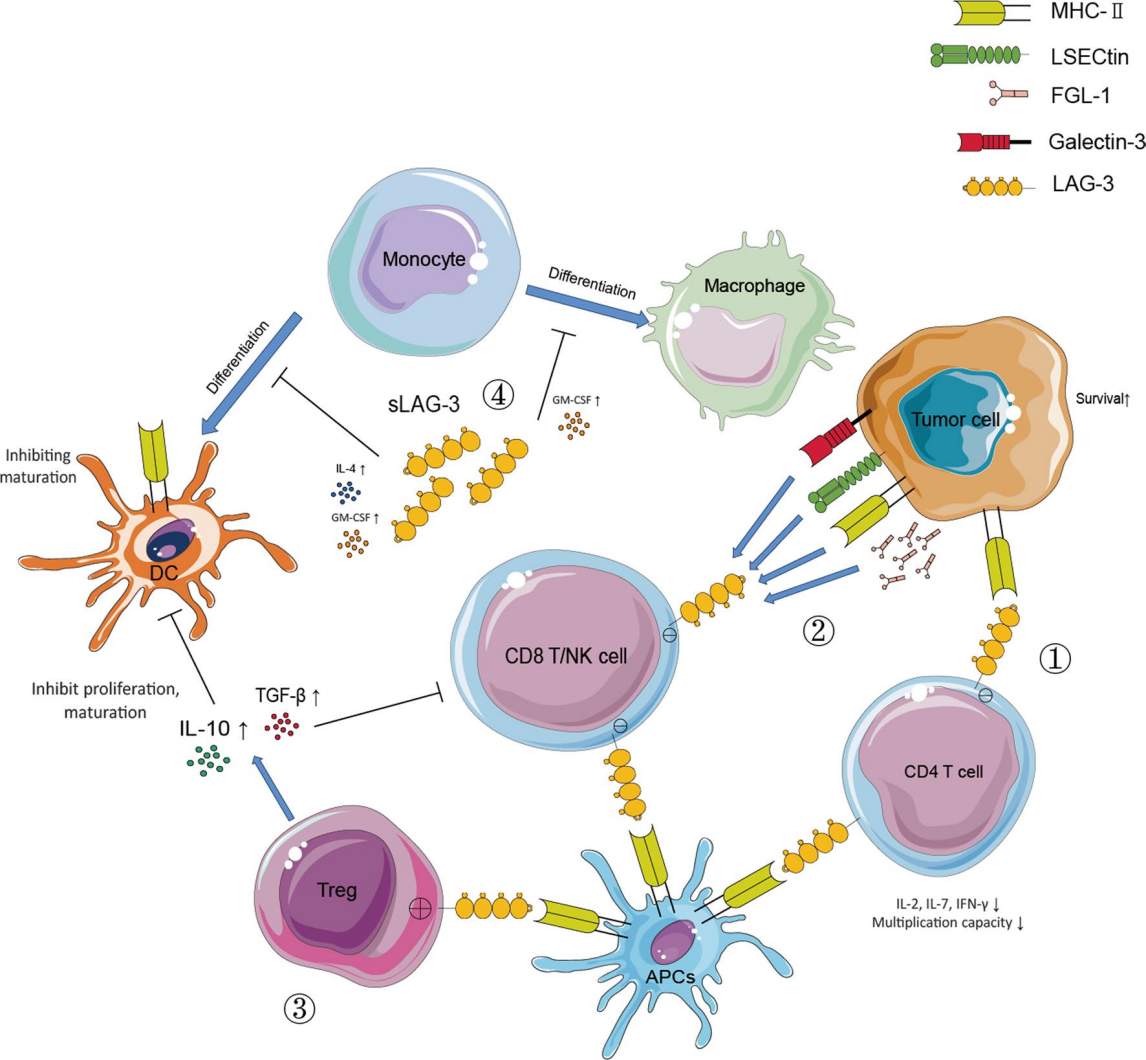
The immunosuppression mechanisms underlying LAG-3 action in the TME. ① The interaction of LAG-3 and MHC-II between CD4 and tumor cells inhibits the proliferation and cytokine secretion of CD4 T cells, and the downstream MHC-II signal may support the survival of tumor cells. ② The interaction of LAG-3 and Galectin-3/LSECtin/FGL-1 between CTL/NK cells and the TME compartment inhibits the proliferation and cytotoxicity of CTL/NK cells. ③ The interaction of LAG-3 and MHC-II between Tregs and tumor cells/DCs enhances the stability and immunosuppression capacity of Tregs. On the other hand, the maturation and immunostimulatory capacity of DCs are impaired by MHC-II downstream signaling. ④ The TME contains soluble LAG-3 (sLAG-3), which can impair the antigen-presenting function of monocyte-derived DCs (mDCs) or even inhibit the differentiation of monocytes into DCs

Here we summarize the immunosuppression mechanisms underlying LAG-3 expression in the TME as follows: 1) The interaction of LAG-3 and MHC-II between CD4 and tumor cells inhibits the proliferation of CD4 T cells and cytokine secretion from these cells; moreover, the signaling downstream of MHC-II supports the survival of tumor cells [85], (2) The interaction of LAG-3 and MHC-II between Treg and tumor cells/DCs may enhance the stability and immunosuppression capacity of the Tregs; however, the maturation and immunostimulatory capacity of DCs may be impaired by MHC-II downstream signaling [26, 28, 86], (3) The interaction of LAG-3 and MHC-II between Treg and tumor cells/DCs may enhance the stability and immunosuppression capacity of the Tregs; however, the maturation and immunostimulatory capacity of DCs may be impaired by MHC-II downstream signaling [31], (4) sLAG-3 in the TME impaired the antigen-presentation function of monocyte-derived DCs (mDCs) in the TME or even inhibit the differentiation of mDCs [87] (Fig. 2). Therefore, blocking LAG-3 may be an effective strategy to enhance antitumor T cell responses. The effects of targeting LAG-3 in melanoma were first reported in an early clinical trial in 2013.

### TIM-3

In cancer patients, TIM-3 overexpression can be detected on most immune cells, particularly antigen-specific CD8^+^ T cells, CD4^+^ T cells, and NK cells [46, 88]. TIM-3 coexpression with PD-1 is frequently found on immune cells of peripheral blood and bone marrow and on TILs from tumor patients, and this expression is correlated with decreased T cell proliferation and cytokine production, resulting in immune cell dysfunction and tumor immune escape [89–92].

In addition to expression on immune cells, TIM-3 is expressed on a number of tumor cells [42, 62, 93, 94]. In 2010, Kikushige Y et al. first reported TIM-3 on the surface of leukemia stem cells (LSCs) but not on hematopoietic stem cells (HSCs) [41]; Since this report, TIM-3 has been thought of as a biomarker for acute myeloid leukemia (AML) stem cells as well as a target for treatments directed against myeloid leukemia stem cells for patients with AML and myelodysplastic syndromes (MDS) [95, 96]. Further study has demonstrated that TIM-3 is expressed on endothelial cells, but in this context, it does not function as a Gal-9 receptor but rather interacts with melanoma cells to trigger the NF-κB signaling pathway, promoting cell proliferation and reducing the apoptosis rate [62]. Furthermore, TIM-3 was also reported to be expressed on tumor cells from patients with NSCLC, HCC, and GC [42, 46, 88].

Most studies indicated that overexpression of TIM-3 either on immune cells or tumor cells is associated with poor OS for patients, such as patients with GC [88], and high-risk groups, showing poor prognosis, and lower complete response (CR) rates following induction chemotherapy in patients with AML as well as high-risk patients with B cell acute lymphoblastic lymphoma (B-ALL) relapse after allogeneic hematopoietic stem cell transplantation (allo-HSCT) [97–100]. Moreover, a Phase I trial (NCT02573363) demonstrated that higher TIM-3/Gal-9 expression was associated with chemotherapy resistance in patients with AML [101].

**Fig. 3:**
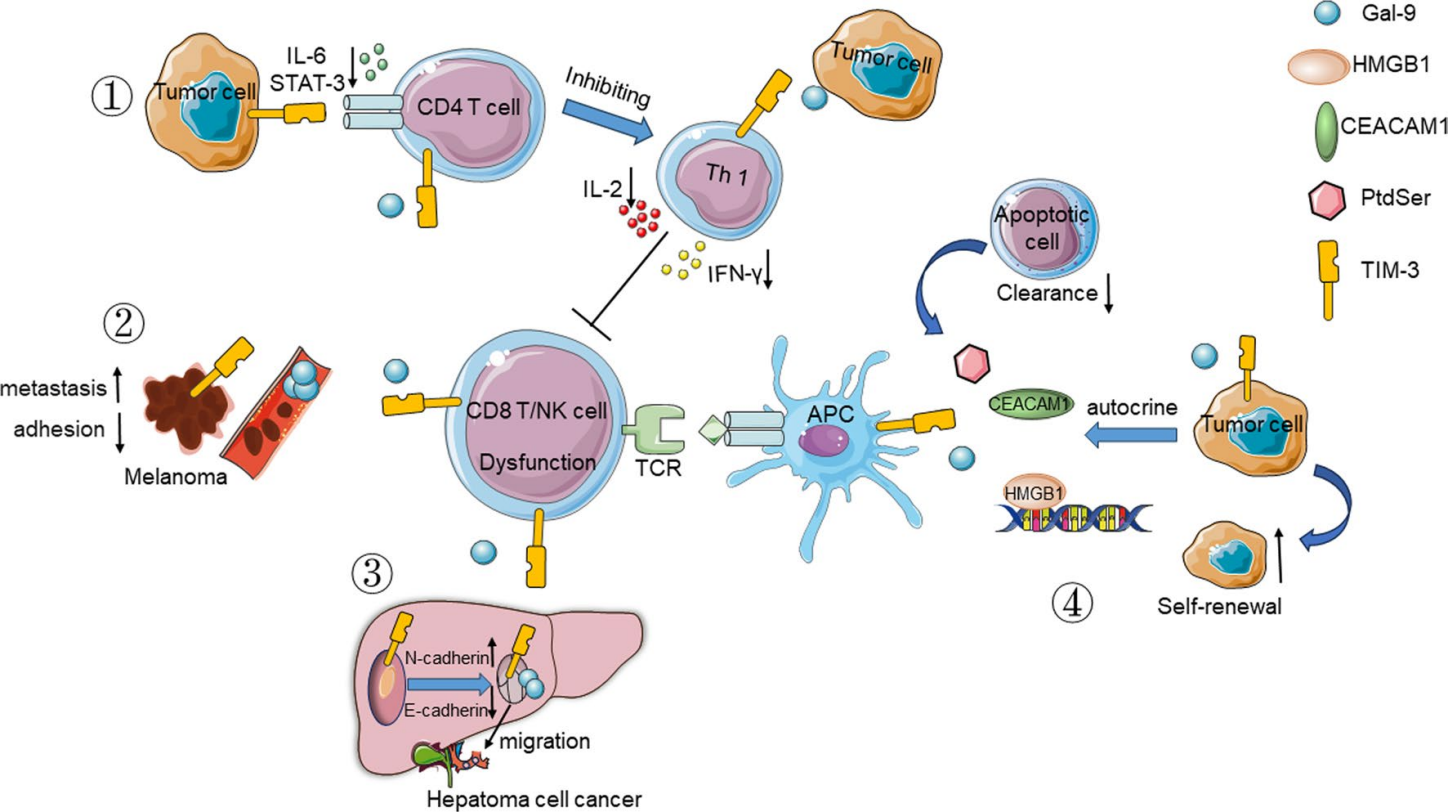
The immunosuppression mechanisms underlying TIM-3 action in the TME. ① Inhibition of CD4 + T cell activation via the IL-6-STAT3 pathway, preventing Th1 polarization and promoting tumor occurrence, growth, and metastasis. ② Expression of TIM-3 on melanoma cells can reduce the adhesion ability of tumor cells and promote their survival. ③ Expression of TIM-3 on HCC cells regulate the epithelial-mesenchymal transition (EMT) by reducing E-cadherin and upregulating N-cadherin expression, which increases the migration and invasion of HCC cells. ④ TIM-3^+^ tumor cells sustain their own survival and self-renewal via the autocrine action a variety of TIM-3 ligands and avoiding recognition and clearance by immune cells

Several studies have demonstrated that TIM-3 expression in tumors may contribute to cancer cell immune escape via different mechanisms, including (1) inhibiting CD4^+^ T cell activation via the IL-6-STAT3 pathway, thereby preventing Th1 polarization and promoting tumor occurrence, growth, and metastasis [42, 63], (2) reducing the adhesion of tumor cells and promoting the survival of melanoma cells [39, 60]. (3) regulating the epithelial-mesenchymal transition by reducing E-cadherin and upregulating N-cadherin expression, which increases HCC cell migration and invasion rates [46], (4) by autocrine signaling mediated through TIM-3 binding ligands, including Gal-9, thereby enabling these cells to avoid recognition and clearance by immune cells to sustain their survival and self-renewal [102–106]. Blocking TIM-3 has been explored as a therapeutic strategy in a number of clinical trials since 2015. The immunosuppressive mechanisms of TIM-3 in the TME are depicted in Fig. 3.

### TIGIT

TIGIT upregulation was identified in several kinds of solid tumors and leukemia, including melanoma, NSCLC, GC, AML, and multiple myeloma [8, 57, 107–111]. In melanoma patients, CD8^+^ TILs highly expressed TIGIT together with PD-1, this high-TIGIT expression was consistent with that observed in NSCLC patients. TIGIT^+^ CD8^+^ T cells from patients with AML, GC, or multiple myeloma showed reduced cytokine production, high susceptibility to apoptosis, and significantly reduced proliferation and killing ability. In addition, CD8^+^ T cells in patients with AML or multiple myeloma with high-TIGIT expression expressed lower levels of CD226. Moreover, the increased expression of TIGIT on CD8^+^ T cells has been related to poor prognosis during leukemia relapse after allo-HSCT and in advanced GC patients [111, 112].

In addition to its expression on TILs, TIGIT is expressed on NK cells. In human CC NK cells, the expression of TIGIT in intratumoral areas was significantly higher than that in the peritumoral area, and the expression of TIGIT on CD8^+^ T cells in the intratumoral area was not significantly different from that in the peritumoral area. In a variety of tumor bearing mice models (B16 melanoma, CT26 CC, 4T1 BC lung metastasis model mice, among others), TIGIT was more specifically associated with tumor progression in NK cells than in other cells, and TIGIT^+^ NK cells acquired an exhaustion phenotype, with reduced effector function and antitumor potential. Lack of TIGIT expression in NK cells in vivo retarded tumor growth, and blockade of TIGIT action via mAb reversed antitumor NK cell exhaustion in multiple tumor models, resulting in increased overall host survival [113]. TIGIT is commonly expressed on Tregs. TIGIT^+^ Tregs acquired both highly activated and suppressed phenotypes in tumor tissues [114]. For example, in bladder cancer, TIGIT ^+^ Tregs accumulated around cancer tissues, promoted cancer cell metastasis and suppressed the antitumor immune response by promoting IL-32 expression in Tregs [115].

Recently, a study showed that TIGIT is expressed on human memory B cells and controls the immune response by directly acting on T cells and blocking the proinflammatory function of dendritic cells, thereby inhibiting Th1-, Th2-, Th17- and CXCR5^+^ICOS^+^ T cell responses while promoting the immune regulatory function of the T cells [116]. All these studies suggest that the increased expression of TIGIT in the TME leads to immune escape of tumor cells, thereby affecting the development and progression of tumors and the prognosis of patients.

**Fig. 4:**
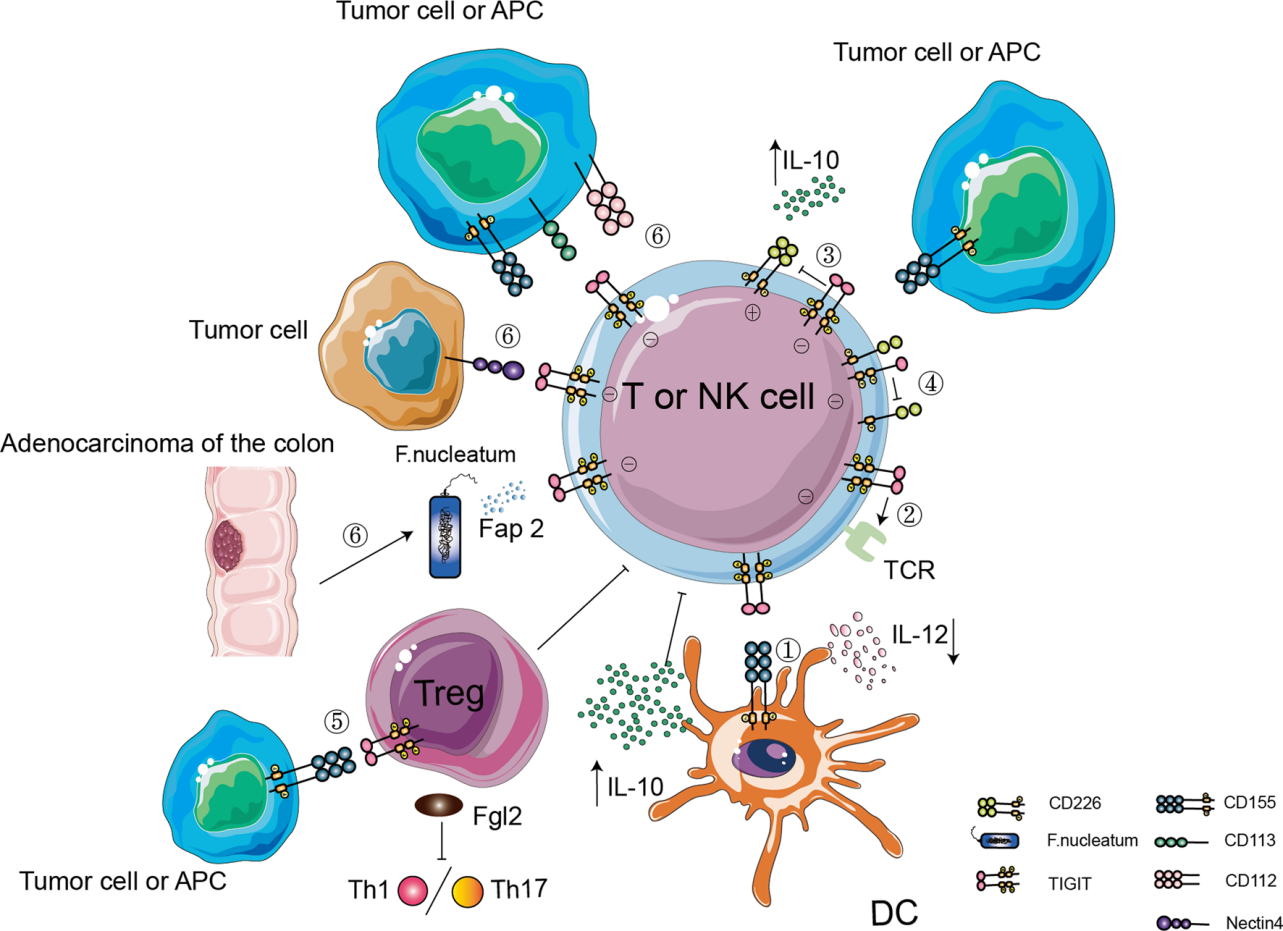
The immunosuppression mechanisms underlying TIGIT action in the TME. ① TIGIT binds to D155 expressed on DCs, making DCs tolerant, resulting in increased IL-10 secretion and decreased IL-12 production. ② TIGIT acts directly on T cells independent of APCs by weakening the activation signal mediated by TCR, thereby inhibiting the T cell response ③ TIGIT and CD226 are coexpressed on T cells, and TIGIT competes with CD226 for binding their common ligand CD155, thereby directly inhibiting the T cell response and increasing the secretion rate of IL-10. ④ TIGIT directly binds to CD226 and prevents the dimerization of CD226, thereby preventing the binding of CD155 to CD226. ⑤ TIGIT expressed on Tregs binds to CD155 expressed on tumor cells or APCs, resulting in increased secretion of the Fgl2 protein, thereby inhibiting Th1/Th17 cell differentiation. ⑥ TIGIT binds to ligands and transmits inhibitory signals directly to T cells and NK cells through its cytoplasmic tail

TIGIT has been shown to potentially suppress innate and adaptive immunity through the following mechanisms: (1) TIGIT acts on DCs by binding to its ligand CD155, promoting the formation of immune tolerogenic DCs and indirectly hindering the function of T cells [51]. (2) TIGIT can act directly on the T cells by attenuating TCR driven activation signals, which is independent of the APC directed inhibition of T cell responses [117]. (3) TIGIT directly inhibits T cell function by competing with CD226 to bind their common ligand CD155, reducing IL-2 and IFN-γ production and increasing the production of IL-10, thereby exerting immunosuppressive effects [118]. (4) TIGIT can directly bind to CD226 in cis, which disrupts the binding of CD226 to CD155 [119]. (5) TIGIT is enriched in Tregs and enhances the immunosuppressive function of Tregs through its effects on an exogenous pathway. TIGIT interferes with selective Treg-mediated suppression of proinflammatory Th1 and Th17 cells, but not Th2 cells, by inducing the secretion of the soluble effector molecule fibrin-like protein-like protein 2 [114, 120]. (6) Furthermore, TIGIT binds to its ligands and transmits inhibitory signals directly to T cells and NK cells through its cytoplasmic tail [121] (Fig. 4). Hence, blocking TIGIT activity is an effective approach to resolve the exhaustion of immune cells and restore their antitumor function. Therapies blocking TIGIT have been explored in a number of clinical trials since approximately 2016.

## Preclinical studies evaluating therapies targeting LAG-3, TIM-3, and TIGIT in solid tumors and leukemia

Several preclinical studies have demonstrated that targeting LAG-3, TIM-3, or TIGIT restores T cell function and inhibits tumor progression, and most studies indicated that coinhibition of different ICPs may effectively enhance antitumor activity.

### LAG-3

Increasing the expression of LAG-3 and LAG-3^+^ immune cells in patients with solid tumors or leukemia has been associated with tumor progression, poor prognosis, and unfavorable clinical outcomes, strongly indicating that LAG-3 contributes to immune escape by tumor cells. These findings are similar to those reported for PD-1, but different mechanisms may be involved. Therefore, LAG-3 has been proposed as a promising therapeutic target for cancer immunotherapy based on results from in vitro and in vivo animal model studies.

Recently, strategies targeting LAG-3 have mainly includes sLAG-3-Ig and anti-LAG-3 antagonistic antibodies. LAG-3 antagonistic Abs can directly bind to LAG-3 molecules, block the interaction between ligands and LAG-3, and down-regulate the inhibitory effect of LAG-3 on the immune system. sLAG-3-Ig (such as IMP321) is composed of the Fc part of the human antibody and the four extracellular domains of LAG-3, which can target the MHC-II molecules on APCs and activate APCs to activate other immune cells, including T cells [30].

Shortly after it was generated for use in biochemical and functional studies, a sLAG-3-Ig fusion protein was studied in vivo in murine tumor models. For example, in an HNSCC mouse model with overexpressed LAG-3 on CD4^+^ and CD8^+^ T cells and Tregs, administration of LAG-3-Ig retarded tumor growth in a manner associated with an enhanced systemic antitumor response; specifically, LAG-3-Ig potentiated the cytotoxicity of CD8^+^ T cells and reduced the population of immunosuppressive cells [73]. sLAG-3-Ig mediated tumor control and regression in mice bearing RCC, sarcoma, or BC. sLAG-3-Ig upregulates the expression of costimulatory molecules and increases IL-12 expression in DCs [122]. These phenotypic changes result in an enhanced sLAG-3-Ig-induced DC maturation, which leads to Th1 cell responses and increases the production of IFN-γ in responding T cells. Based on these findings, sLAG-3-Ig has been proposed to function as an adjuvant that likely can potentiate a response to a vaccine. This proposal was realized, as sLAG-3-Ig has been shown to markedly enhance the CD8^+^ T cell response to a soluble antigen vaccine (ovalbumin) as well as the humoral response to a particulate antigen (hepatitis B surface antigen) in mice [123]. This adjuvant effect has been extended to cancer vaccines as well.

In contrast to the effects of sLAG-3-Ig, anti-LAG-3 mAbs mainly block the LAG-3/MHC-II interaction to restore immune cell function. High expression of LAG-3 in patients with CLL has been associated with poor cytogenetics and poor prognosis. After anti-LAG-3 mAb treatment in vitro, peripheral blood mononuclear cells from CLL patients eliminated leukemic cells and exhibited restored NK and T cell-mediated responses [124]. In soft tissue sarcoma model mice, LAG-3 blockade decreased tumor growth and enhanced the secretion of IFN-γ by CD8^+^ and CD4^+^ T cells [69]. However, due to the limited efficacy of the anti-LAG-3 mAb administered alone, it is generally used in combination with other ICIs, including CTLA-4 inhibitors or PD-1 inhibitors, increasing their efficacy synergistically.

It has been shown that LAG-3 functions in concert with PD-1 to suppress antitumor immunity. Coexpression of LAG-3 and PD-1 on tumor-infiltrating CD4^+^ and CD8^+^ T cells and the profound therapeutic effects of coblockers or the genetic deletion of LAG-3 and PD-1 have been observed in various model mice of tumors, including B16 melanoma, MC38 colon adenocarcinoma, Sa1N fibrosarcoma, IE9mp1 ovarian cancer (OC), Em-TCL1 CLL, and recurrent melanoma [75, 125–127]. The blockade of both LAG-3 and PD-1 augmented the proliferation and cytokine production of tumor-infiltrating CD8^+^ T cells after ex vivo stimulation with the tumor-associated antigen NY-ESO-1 in OC cells [128]. Therefore, targeting multiple ICPs simultaneously has become a promising therapeutic strategy.

In 2019, Fianlimab (REGN3767), a fully human IgG4 mAb that targets LAG-3, was developed. This mAb binds human and monkey LAG-3 with high affinity, and specificity blocks the interaction between LAG-3 and MHC-II. In an engineered T/APC bioassay, fianlimab, alone or in combination with cemiplimab (REGN2810, a human anti-PD-1 mAb), blocked inhibitory signaling to T cells mediated by hLAG-3/MHC-II in the presence of PD-1/PD-L1. In humanized *PD-1xLAG-3* knock-in mice, treatment with cemiplimab and fianlimab showed increased efficacy and enhanced the amount of proinflammatory cytokines secreted by tumor-specific T cells compared with the effect of fianlimab alone [129]. Another study described the profound effects of combined inhibition of LAG-3 (BI 754111) and PD-1 (ezabenlimab) in an in vitro model of antigen-exposed memory T cells expressing PD-1 and LAG-3. IFN-γ secretion was increased as high as 13.2-fold compared to that of the isotype control in the BI 754111 plus ezabenlimab group and was increased 1.8-fold and 6.9-fold in the BI 754111 and ezabenlimab monotherapy groups, respectively [130]. These results supported the clinical investigation of a combination treatment to inhibit PD-1 and LAG-3. Clinical trials involving anti-LAG-3 mAb treatment have been conducted since 2006.

### TIM-3

Similar to the effects of PD-1 blockade, the effects of blocking TIM-3 in vitro and ex vivo experiments demonstrated that it can improve cytotoxicity and IFN-γ release by both TILs and NK cells in RCC, melanoma, lung adenocarcinoma, and OC contexts [131].

The first study demonstrating that blocking the TIM-3/Gal-9 pathway by an anti-TIM-3 mAb increases the activation and numbers of macrophages in a mouse model of autoimmune disease was reported in 2002 [132]. Further study using a TIM-3 fusion protein confirmed that the TIM-3–TIM-3 ligand pathway may inhibit the expansion and effector functions of Th1 cell populations and may be essential for tolerance induction in Th1 cells [133]. Later, lower immune tolerance mediated via a reduction in the expansion of myeloid-derived suppressor cells was demonstrated in mice with TIM-3^−/−^4T1 mammary adenocarcinoma after TIM-3 Ig fusion protein treatment [134]. In 2010, anti-TIM-3 mAbs were first used in both CT26 CC mice and mice bearing B16 melanoma, but they demonstrated little effect. However, significant antitumor effects were demonstrated when TIM-3-Ig was administered in combination with an anti-PD-L1 mAb [135]. Similarly, studies demonstrated that anti-TIM-3 (5D12 clone) alone was not effective in reducing tumor growth in CC (CT26 and MC38) model mice, while blockade of both CEACAM-1 and PtdSer via TIM-3 showed greater efficacy [102, 136]. In contrast, other experiments have shown that anti-TIM-3 mAb alone exerted an antitumor effect on WT3 sarcoma, a MC38 tumor, and B16 melanoma models [41, 131, 137].

As previously described, TIM-3 is expressed on myeloid LSCs; thus, Kikushige Y et al. successfully reconstructed an AML model using TIM-3^+^ AML cells in immunodeficient mice and established the first anti-human TIM-3 mouse IgG2a mAb that did not disrupt the reconstitution of normal human HSCs but blocked LSCs [41]. TIM-3^+^ AML LSCs secrete the ligand Gal-9 in an autocrine manner, activating the NF-κB and β-catenin pathways to increase survival and self-renewal [106]. In addition, anti-TIM-3 alone can inhibit leukemia cell proliferation in AML model mice [41, 131, 137]. However, additional findings indicate the necessity of targeting multiple coinhibitory ICPs rather than targeting TIM-3 alone to maximize therapeutic efficacy in the context of AML and solid tumors in mice [131, 137].

The antitumor effects by targeting TIM-3 may be required under certain conditions; for example, in the cases of IFN-γ-producing CD8^+^ and CD4^+^ T cells and when the ratio of tumor-infiltrating CD8^+^:CD4^+^ T cells is high [131]. Moreover, in a CT26 subcutaneous tumor model, blocking TIM-3 was effective only before the appearance and accumulation of a significant number of TIM-3^+^PD-1^+^ T cells [131]. In contrast, many preclinical studies have revealed that TIM-3 is upregulated in immunotherapy resistance, and high expression of TIM-3 on T cells may be related to adaptive resistance to anti-PD-1 or anti-CTLA-4 treatment. Blocking TIM-3 can increase the antitumor effects of anti-PD-1 or anti-CTLA-4 immunotherapy. TIM-3 and PD-1 coblockers increased antitumor immune responses and tumor growth-reducing efficacy in melanoma and GC mouse models [60, 135, 138–141]. The anti-TIM-3 mAb has been shown to increase the resistance of anti-PD-1 therapy in mouse models of lung adenocarcinoma with genetically engineered EGFR and extended the median survival from 5 to 11.9 weeks. The effects not only included enhanced T cell function following anti-PD-1 mAb failure but also decreases in the levels of tumor-promoting cytokines, such as IL-6 and progranulin [138]. Overall, targeting TIM-3 can be considered a strategy to overcome resistance to anti-PD-1 therapy. In addition, several attempts have been made to investigate the synergistic efficacy of TIM-3 inhibitor combined with chemotherapy or radiotherapy and anti-PD-1/PD-L1 therapy in tumor model mice [139, 142, 143]. For example, adding anti-TIM-3 mAbs to an anti-PD-1 mAb therapy regimen prolonged median survival from 33 to 100 days and increased OS from 27.8 to 57.9% in model mice with glioblastoma [142]. TIM-3 blocker has been evaluated in several clinical trials since 2015.

### TIGIT

Because TIGIT hinders multiple stages of antitumor immunity, it is abnormally expressed in several cancer types and associated with poor clinical outcome. Numerous preclinical studies have evaluated TIGIT blockade immunotherapy in the contexts of various solid tumors and leukemia.

TIGIT blockade was first observed in a study that showed that deletion of the TIGIT gene in mice significantly enhanced the cytotoxic effects of NK cells and CD8^+^ T cells against tumor cells [144]. In addition, TIGIT modulate the suppressive activity of Tregs, thereby promoting tumor growth in B16F10 melanoma model mice, and these findings were further demonstrated in TIGIT knockout mice. In OC model mice, blocking TIGIT significantly reduced tumor growth and the proportion of CD4^+^ Tregs and increased the survival rate [145, 146]. Overall, TIGIT blockade can enhance NK cell cytotoxicity and CD4^+^ and CD8^+^ T cell activation, inhibit Treg activity, and improve antitumor effects in vitro and in vivo in mouse models.

TIGIT expression is closely associated with PD-1 on T cells in patients with solid tumors or leukemia [147–150]. Anti-TIGIT mAbs alone or in combination with anti-PD-L1 mAbs synergistically exerted their effects in a CT26 colorectal cancer model mice [57]. Treatment with a combination of anti-PD-1 and anti-TIGIT mAbs more effectively controlled tumor growth [57]. In ex vivo experiments, combination treatment including atezolizumab (anti-PD-L1 mAb) and tiragolumab (anti-TIGIT mAb) restored the functionality of TILs from colorectal cancer patients [151].

In contrast, the immunosuppressive effects of TIGIT can be leveraged for acute graft-versus-host disease (GVHD) therapy after allo-HSCT. A study using a TIGIT-Fc fusion protein, which exerted immunosuppressive effects by binding to CD155 on DCs, demonstrated that TIGIT-Fc delayed the onset of GVHD symptoms and increased survival in model mice with acute GVHD. TIGIT inhibition can also be utilized in transplant immunotherapy because it enhances the activity and function of graft immune cells with disease relapse after allo-HSCT [152]. Therefore, targeting TIGIT holds clear clinical potential as a cancer treatment, and various TIGIT-targeting mAbs have been evaluated in clinical trials for the treatment of solid tumors since 2016.

## Clinical trials of anti-LAG-3, anti-TIM-3, and anti-TIGIT mAbs

Several clinical trials have been established to evaluate anti-LAG-3, anti-TIM-3, or anti-TIGIT mAbs as different tumor therapies. Most of these trials are in Phase I/II for patients with advanced and metastatic cancers; the final results have not been reported. Multiple Phase III trials have shown positive results, and on 2022, both of the FDA and the European Medical Agency (EMA) approved Opdualag (a fixed-dose combination of the anti-LAG-3-blocking mAb relatlimab and the anti-PD-1-blocking mAb nivolumab) for the treatment of adults and children 12 years of age or older with unresectable or metastatic melanoma [7].

### Anti-LAG-3 mAbs

A number of anti-LAG-3 mAbs have been developed in the past year, and some are currently being evaluated in clinical trials as cancer immunotherapies (Table 3). These trials have been completed, are underway, or are recruiting participants (ClinicalTrials.gov). Two types of inhibitors have been developed for LAG-3-targeting therapies: anti-LAG-3 mAbs and LAG-3-bispecific antibodies (BsAbs). In this section, we describe the clinical trials in which anti-LAG-3 mAbs are being evaluated.

**Table 3:** Anti-LAG-3 mAbs and associated clinical trials in cancer

Clinical trial identifier	Phase	Start date	Status	Cancer type (population, N)	Interventions and Combination	Target	Primary Outcome Measures	Secondary Outcome Measures
NCT0206176	I/II	Mar 13, 2014	Completed	HMs, * N* = 106	Relatlimab	LAG-3	AEs, SAEs	Cmax, Tmax
NCT02720068	I	May 2, 2016	Active, not recruiting	Neoplasms, * N* = 576	Favezelimab	LAG-3	DLTs, AEs	ORR
NCT03005782	I	Nov 7, 2016	Active, not recruiting	Malignancies, * N* = 333	REGN3767	LAG-3	Cmax, Tmax	RECIST
NCT04566978	I	Sep 11, 2020	Recruiting	DLBCL, * N* = 20	89Zr-DFO-REGN3767	LAG-3	Biodistribution	–
NCT03489369	I	May 8, 2018	Completed	Metastatic Cancer Solid Tumor Lymphoma, * N* = 15	Sym022	LAG-3	AEs	OR, SD, TTP
NCT02195349	I	Jul 30, 2014	Completed	Psoriasis, * N* = 67	GSK2831781	LAG-3	PCI, AEs, SAEs	PASI, PGA
NCT02460224	I/II	Jun 17, 2015	Completed	Advanced Solid Tumor, * N* = 490	LAG525	LAG-3	DLTs, ORR	AEs, RDI
NCT03250832	I	Aug 8, 2017	Completed	Neoplasms, * N* = 111	TSR-033	LAG-3	SAEs, TEAEs	AUC, Cmax
NCT03538028	I	Jun 18, 2018	Completed	MSI-High Endometrial Cancer, CCA, GC, * N* = 22	INCAGN02385	LAG-3	TEAEs	Cmax, Tmax, ORR
NCT02935634	II	Nov 29, 2016	Completed	AGC, * N* = 190	Relatlimab + Nivolumab	LAG-3 PD-1	ORR, DOR	AEs
NCT02488759	I/II	Oct 13, 2015	Completed	Various Advanced Cancers, *N* = 578			AEs, ORR	DOR, OS
NCT02996110	II	Feb 2, 2017	Completed	Advanced Cancer, * N* = 182			ORR, DOR	AEs
NCT02519322	II	Feb 2, 2016	Completed	CM, MM, OM, * N* = 53				OS
NCT02061761	I/II	Mar 13, 2014	Completed	HMs, * N* = 106			AEs	Cmax, Tmax
NCT03310619	I/II	Nov 28, 2017	Completed	NHL, DLBCL, FL, * N* = 62	JCAR017 + Relatlimab	LAG-3 CD19	DLTs	AEs, PFS
NCT04112498	I	Oct 1, 2019	Completed	Cancer, * N* = 24	Relatlimab + rHuPH20 + Nivolumab	LAG-3 CD38 PD-1	Cmax, SAEs	AEs
NCT04626518	II	Dec 17, 2020	Recruiting	ccRCC, * N* = 370	Favezelimab + Pembrolizumab	LAG-3 PD-1	DLTs, AEs	DOR, PFS
NCT05342636	I/II	Jul 27, 2022	Recruiting	ESCC, * N* = 120			DLTs, AEs	PFS, DOR
NCT04938817	I/II	Aug 19, 2021	Active, not recruiting	SCLC, * N* = 80			AEs, ORR	PFS, DOR
NCT02625961	II	Feb 10, 2016	Recruiting	BC, * N* = 320			DFS	DOR
NCT05845814	I/II	Jul 10, 2023	Recruiting	mUC, UN, * N* = 390			ORR, AEs	PFS, DOR
NCT03516981	II	Oct 1, 2018	Active, not recruiting	Advanced NSCLC, * N* = 318			ORR	PFS, OS
NCT02720068	I	May 2, 2016	Active, not recruiting	Neoplasms, * N* = 576			DLTs, AEs	ORR
NCT03598608	I/II	Oct 17, 2018	Recruiting	NHL, DLBCL, * N* = 174			DLTs, AEs	ORR
NCT05508867	III	Oct 18, 2022	Recruiting	NHL, * N* = 360			PFS	OS, ORR
NCT05064059	III	Nov 10, 2021	Active, not recruiting	CRC, * N* = 432			OS	PFS, ORR
NCT05600309	III	Jun 14, 2022	Recruiting	CRC, * N* = 94			OS	PFS, ORR
NCT05137054	I	Aug 17, 2022	Recruiting	MM, * N* = 317	REGN3767 + Linvoseltamab	LAG-3 BCMA CD3	DLTs, TEAEs	ORR, DOR
NCT01042379	II	Mar 1, 2010	Recruiting	BC, * N* = 5000	REGN3767 + Cemiplimab	LAG-3 PD-1	pCR	RCB, RFS
NCT03916627	II	Jul 23, 2019	Recruiting	NSCLC, HCC, HNSCC Carcinoma, * N* = 73CLC			MPR, STN	ORR, OS
NCT05785767	II/III	Jun 16, 2023	Recruiting	Advanced NSCLC, * N* = 850			ORR, OS	TEAEs, SAEs
NCT05352672	III	Jul 14, 2022	Recruiting	Melanoma, * N* = 1590			PFS	OS, DCR
NCT03005782	I	Nov 7, 2016	Active, not recruiting	Malignancies, * N* = 333			AUC, CL	ORR
NCT03311412	I	Nov 20, 2017	Completed	Metastatic Cancer, Solid Tumor, Lymphoma, * N* = 89	Sym022 + Sym021 + Sym023	LAG-3 PD-1 Tim-3	AEs	OR, SD
NCT04641871	I	Oct 12, 2020	Active, not recruiting	Metastatic Cancer, Solid Tumor, * N* = 148	Sym022 + Sym021	LAG-3 PD-1	ORR, SAEs	Cmax, AUC
NCT03484923	II	Sep 10, 2018	Completed	Melanoma, * N* = 196	LAG525 + Spartalizumab	LAG-3 PD-1	ORR	OS, PFS
NCT03499899	II	Jul 2, 2018	Completed	TNBC, * N* = 88	LAG525 + PDR001 + Carboplatin	LAG-3 PD-1	ORR	CBR, DOR
NCT02460224	I/II	Jun 17, 2015	Completed	Advanced Solid Tumors, * N* = 490	LAG525 + PDR001	LAG-3 PD-1	DLTs, ORR	AEs, Cmax
NCT03365791	II	Jan 24, 2018	Completed	SCLC, GAC, EAC, CRPC Adenocarcinoma, * N* = 76			CBR	ORR, TTR
NCT03250832	I	Aug 8, 2017	Completed	Neoplasms, * N* = 111	TSR-033 + Dostarlimab + mFOLFOX6 + FOLFIRI + Bevacizumab	LAG-3 PD-1 VEGF-A	SAEs, TEAEs	AUC, Cmax
NCT04463771	II	Jan 26, 2021	Recruiting	UCEC, * N* = 300	INCAGN02385 + INCMGA00012	LAG-3 PD-1	ORR	CR, OS
NCT04370704	I/II	Jul 27, 2020	Recruiting	Melanoma, * N* = 146	INCAGN02385 + INCAGN02390 + INCMGA00012	LAG-3 PD-1 Tim-3	TEAEs, ORR	DCR
NCT05287113	II	Nov 14, 2022	Recruiting	HNSC, * N* = 162	INCAGN02385 + Retifanlimab	LAG-3 PD-1	PFS	ORR, DOR, etc

Notes: AEs: Adverse Events, AGC: Advanced Gastric Cancer, AUC: Area Under the Plasma Concentration Versus Time Curve, BC: Breast Cancer, CRPC: Castration Resistant Prostate Cancer, CCA: Cervical cancer, ccRCC: Clear Cell Renal Cell Carcinoma, CL: Clearance, CBR: Clinical Benefit Rate, CRC: Colorectal Cancer, CR: Complete Response, CM: Cutaneous Melanoma, DFS: Disease-free survival, DLBCL: Diffuse Large B cell Lymphoma, DCR: Disease Control Rate, DLTs: Dose-limiting Toxicities, DOR: Duration of Response, EAC: Esophageal Adenocarcinoma Cancer, ESCC: Esophageal Squamous Cell Carcinoma, FL: Follicular Lymphoma, GAC: Gastric Adenocarcinoma Cancer, GC: Gastric Cancer, HCC: Hepatocellular Carcinoma Cancer, HNSC: Head and Neck Squamous Cell, HMs: Hematological Malignancies, MPR: Major Pathologic Response, Cmax: Maximum Observed Serum Concentration, mUC: Metastatic Urothelial Carcinoma, MM: Mucosal Melanoma, NHL: Non-Hodgkin Lymphoma, NSCLC: Non-small Cell Lung Cancer, ORR: Objective Response Rate, OR: Objective Response, OM: Ocular Melanoma, OS: Overall Survival, pCR: pathological Complete Response, PGA: Physician Global Assess, PCI: Potential Clinical Importance, PFS: Progression-free Survival, PASI: Psoriasis Area and Severity Index, RFS: Relapse-free Survival, RDI: Relative Dose Intensity, RCB: Residual Cancer Burden, RECIST: Response Evaluation Criteria in Solid Tumors, SAEs: Serious Adverse Events, STN: Significant Tumor Necrosis, SCLC: Small Cell Lung Carcinoma, SD: Stable Disease, Tmax: Time of Maximum Concentration, TTP: Time to Progress, TTR: Time to Response, TEAEs: Treatment-emergent Adverse Events, TNBC: Triple-negative Breast Cancer, UN: Urothelial Neoplasms, UCEC: Uterine Corpus Endometrial Carcinoma, VEGF-A: Vascular Endothelial Growth Factor-A

Relatlimab (BMS-986016) is an anti-LAG-3 fully human IgG4-κ mAb and the first LAG-3 blocker to be clinically developed. Relatlimab binds LAG-3 with high affinity and inhibits its binding to MHC-II. Currently, relatlimab is being evaluated alone or in combination with anti-PD-1/PD-L1 mAbs in Phase I to III trials for patients with solid tumors or leukemia, and it presented good tolerance profile and clinical activity [153]. One important trial for relatlimab evaluation is RELATIVITY-047, a Phase III trial that evaluated the effect of inhibiting both LAG-3 and PD-1 by the combination of relatlimab and nivolumab compared with the effect of nivolumab alone for patients with untreated metastatic or unresectable melanoma (NCT03470922). Blinded independent assessment of the primary end point showed that patients taking relatlimab–nivolumab dual checkpoint inhibitors experienced a PFS that was twice the median PFS and a 25% lower risk of disease progression or death than patients receiving nivolumab alone. The relatlimab–nivolumab cohort showed a slightly greater incidence of adverse events than the nivolumab cohort, but the quality of life measurements was similar. The benefit of relatlimab–nivolumab compared to that of nivolumab was also observed among BRAF-mutant melanoma patients and wild-type individuals. Overall, the trial provided solid data supporting relatlimab–nivolumab as a potential new treatment option for patients with previously untreated metastatic or unresectable melanoma [7]. In June 2022, Bristol Myers Squibb announced the first-in-class dual immunotherapy, relatlimab–nivolumab fixed-dose combination Opdualag, which received accelerated FDA approval for the treatment of metastatic melanoma. This is the first FDA-approved anti-LAG-3 mAb combination therapy, making LAG-3 the third clinical ICI target in the clinic, following PD-1/PD-L1 and CTLA-4.

To date, 65 clinical trials have been established to evaluate relatlimab. In a clinical trial with CLL patients (NCT02061761), relatlimab induced the depletion of leukemia cells in vitro, restored T and NK cell-mediated antitumor responses and promoted T cell production of cytokines such as IL-2. These results provide new insights into the anti-leukemia potential of relatlimab, which may be related to reduced anti-apoptotic signaling in malignant cells and enhanced responses mediated by NK and T cells [124].

Favezelimab (MK-4280) is a humanized IgG4 anti-LAG-3 mAb developed by Merck. Favezelimab treatment increases the production of cytokines, such as IFN-γ, IL-2, IL-8, and TNF-α, and chemokines (CCL4, CXCL10, and CCL22) in Jurkat Clone G10-PD-1 cells (a group of cell lines obtained by coculturing Jurkat and Raji B cell lymphoma lines that express high levels of LAG-3 and PD-1). The expression of CD69, CD44, and CD25 was also upregulated [154]. To date, 15 clinical trials have been established to evaluate favezelimab alone or in combination with other ICIs in different types of tumors. The preliminary results have demonstrated that this drug shows good safety and efficacy and controllable tolerance when administered alone or in combination with other ICIs [155]. The first Phase I/II trial confirmed the safety and effectiveness of pembrolizumab (anti-PD-1) for treating solid tumors [156]. In addition, the combination treatment consisting of favezelimab with pembrolizumab was entered into three Phase III trials for evaluation as a colorectal cancer and Hodgkin lymphoma (HL) treatment (NCT05600309, NCT05064059, and NCT05508867).

Fianlimab (REGN3767) is a fully human, hinge-stabilized, high-affinity IgG4 mAb developed by Regeneron Pharmaceuticals [157, 158]. Fianlimab blocks the interaction between LAG-3 and MHC-II to activate T cells and enhance tumor cell damage mediated by cytotoxic T cells [129]. To date, six clinical trials evaluating fianlimab as a monotherapy or in combination with anti-PD-1 inhibitors in patients with melanoma, NSCLC, lymphoma, and HNSCC have been established.

89Zr-DFO-REGN3767 (a fianlimab tracer) is composed of the anti-LAG-3 mAb fianlimab labeled with a radioactive isotope, the positron-emitter zirconium-89 (89Zr), through a chelator linker [159]. 89Zr-DFO-REGN3767 is currently under evaluation in two clinical trials established to monitor patient response to anti-LAG-3 therapy (NCT04566978 and NCT04706715). The main objective of the clinical trials is to better understand how the body absorbs, distributes, and disposes of 89Zr-DFO-REGN3767 and to identify the best dose and best time to perform a PET scan after injection.

Sym022 is a recombinant, Fc-inert, and fully human mAb developed by Symphogen that blocks LAG-3/MHC-II binding. Similar to the above mentioned mAbs, Sym022 induces T cells to produce cytokines in vitro and inhibits tumor growth in vivo [157]. Three clinical trials have been established to investigate Sym022 alone or in combination with Sym021 (an anti-PD-1 mAb) and Sym023 (an anti-TIM-3 mAb) (NCT03489369, NCT03489369, and NCT03311412). The preliminary data have shown that Sym021 monotherapy is well tolerated and exhibits both immune modulation and antitumor activity, and in combination with Sym022 and Sym023, a synergistic antitumor effect was reported [160]. The data from clinical trials of Sym022 used for patients with advanced solid tumors or lymphomas demonstrated no serious adverse drug reactions after the first and second doses. The third dose caused chest pain in one of three patients, and the fourth dose caused gastrointestinal hemorrhaging, increased lipase levels, and tumor pain in one of the six patients in the trial [161].

INCAGN02385 is an Fc-engineered IgG1κ mAb with the ability to potently block LAG-3 binding with MHC-II. INCAGN02385 increases T cell reactivity to TCR stimulation during monotherapy and in the presence of anti-PD-1/PD-L1 mAbs. INCAGN02385 treatment in cynomolgus monkeys was well tolerated, and a safe pharmacokinetic profile was reported [162]. Clinical studies on the safety and tolerability of INCAGN02385 in patients with certain advanced malignancies have been completed (NCT03538028), and four clinical trials with patients with HNSCC, melanoma, urothelial carcinoma (UC), or endometrial cancer are recruiting (NCT05287113, NCT04370704, NCT04586244, and NCT04463771).

Two other anti-LAG-3 mAbs include Ieramilimab (LAG525) and TSR-033. Leramilimab is a humanized IgG4 mAb that blocks LAG-3 binding to MHC-II. Five clinical trials in different phases have been conducted to evaluate Ieramilimab effectiveness. Four of these trials were completed, and one was terminated. Leramilimab in combination with spartalizumab (anti-PD-1 mAb) was well tolerated and showed initial antitumor activity in patients with mesothelioma and triple-negative BC, neuroendocrine tumors, small cell lung cancer (SCLC), and DLBCL. A clinical benefit rate of 86% was reported for gastrointestinal and pancreatic neuroendocrine tumor cohorts [163, 164]. TSR-033 is a humanized IgG4 mAb that shows high binding affinity for LAG-3 and is a functional antagonist. A preclinical study showed that double blockade of LAG-3 and PD-1 with TSR-033 and TSR-042 increased the total amount and proliferation rate of T cells in model mice harboring humanized NSCLC tumors compared to the effects of TSR-042 monotherapy; these results are consistent with increased antitumor efficacy [165]. TSR-033 is currently being evaluated in two clinical trials in the recruiting phase for the treatment of advanced solid tumors (NCT03250832 and NCT02817633).

### Anti-TIM-3 mAbs

To date, 33 mAbs have been designed to target TIM-3 and for use alone or in combination with other ICIs, chemotherapy agents, targeted therapy drugs, or radiotherapy in clinical trials to evaluate their antitumor activity. The clinical trial details for these drugs are summarized in Table 4.

**Table 4:** Anti-TIM-3 mAbs and associated clinical trials in solid tumors and leukemia

Clinical trial identifier	Phase	Start date	Status	Cancer type (population, N)	Interventions and Combination	Target	Primary Outcome Measures	Secondary Outcome Measures
NCT05738980	Not Applicable	Feb 1, 2023	Recruiting	HCC, * N* = 88	Auto-anti-TIM-3-blocked RAK cells	TIM-3	RFS	OS
NCT03489343	I	May 24, 2018	Completed	Advanced Solid Tumor or Lymphomas, * N* = 24	Sym023	TIM-3	AEs, DLTs	Immunogenicity, OR, SD, TTP
NCT04623892	I	Dec 01, 2020	Unknown	Advanced Solid Tumors, * N* = 50	TQB2618	TIM-3	MTD	Tmax, Cmax, ORR, PFS, DOR, DCR
NCT04823624	II	Sep 2021	Unknown	Lower Risk MDS, * N* = 20	MBG453	TIM-3	ORR,	TEAEs, PFS, OS
NCT03652077	I	Sep 24, 2018	Completed	Select Advanced Malignancies, * N* = 40	INCAGN02390	TIM-3	AEs, Tmax, PAD	Immunogenicity, ORR, DOR, DCR, PFS, Cmax, Tmax
NCT05020912	II	Dec 13, 2021	Completed	HR/vHR MDS, * N* = 20	MBG453 + azacytidine + venetoclax	TIM-3	DLTs, CRR	CR, ORR, PFS, OS
NCT03946670	II	Jun 4, 2019	Active, not recruiting	IM/H/VH-MDS, * N* = 127	MBG453 + HMAs	TIM-3	CRR, PFS	OS, LFS, ORR, DCR
NCT04623216	I/II	Sep 14, 2021	Recruiting	AML/AML MRD + post-aHSCT, * N* = 59	MBG453 + Azacitidine	TIM-3	DLTs, CRR	grade III or IV aGvHD, cGvHD, ADA, Cmax, iR-AEs
NCT04878432	II	Mar 17, 2022	Recruiting	IM/H/VH-MDS, * N* = 90	MBG453 + HMA	TIM-3	TEAEs, SAEs	CRR, OS, PFS, LFS, DOR
NCT04266301	III	Jun 8, 2020	Active, not	IM/H/VH-MDS, CMML-2, * N* = 530	MBG453 + Azacitidine	TIM-3	OS	Safety, CR, etc.
NCT04443751	I	Sep 10, 2020	Recruiting	R/R-AML, HR-MDS, * N* = 42	SHR-1702	TIM-3	MTD, RP2D	Safety, PK, etc.
NCT04150029	II	Sep 1, 2020	recruiting	ND-AML, * N* = 86	MBG453 + HMA + Venetoclax	TIM-3	Safety, DLTs CR rate	CR/CRi rate, OS, etc.
NCT04812548	II	May 31, 2021	Not yet recruiting	HR-MDS, * N* = 76	MBG453 + HMA + Venetoclax	TIM-3	Safety, DLTs, CR rate	ORR, PFS, etc.
CTR20201781	III	Aug 6, 2020	recruiting	HR-MDS, CMML-2, * N* = 100	MBG453 + Azacitidine	TIM-3	OS	Safety, CR, etc.
NCT03680508	II	Dec 19, 2019	Recruiting	HCC, * N* = 42	TSR-022 + TSR-042	TIM-3, PD-1	ORR	irRC, DOR, TTP PFS, OS
NCT03311412	I	Nov 20, 2017	Completed	Advanced Solid Tumor or Lymphomas, * N* = 89	Sym023 ± Sym021	TIM-3, PD-1	AEs, DLTs	Immunogenicity, OR, SD, TTP
NCT03099109	Ia/Ib	Apr 12, 2017	Active, not recruiting	advanced relapsed/refractory solid tumors, * N* = 275	LY3300054 ± LY3321367	TIM-3, PD-L1	DLTs	PK, ORR, PFS, DOR, TTP, DCR
NCT02608268	I-Ib/II	Nov 23, 2015	Terminated	Advanced Malignancies, * N* = 252	PDR001 ± MBG453	TIM-3, PD-1	Safety, ORR, DLTs	BOR, Cmax, DOR, OS, PFS, irRC
NCT02817633	I	Jul 8, 2016	Recruiting	Advanced Solid Tumors, * N* = 475	TSR-042 ± TSR-022	TIM-3, PD-1	DLTs, AEs, SAEs, TEAEs, irAEs, ORR	ORR, DOR, DCR, PFS, OS
NCT04641871	I	Oct 12, 2020	Active, not recruiting	BTC, ESCC, * N* = 148	Sym021 + Sym023 + irinotecan	TIM-3, PD-1	ORR, AEs, SAEs	Cmax, Tmax, ORR, DOR, DCR, PFS, OS
NCT05645315	Ib	Apr 28, 2022	Recruiting	Advanced Solid Tumors, * N* = 127	TQB2618 + TQB2450	TIM-3, PD-L1	ORR, DLTs	Immunogenicity, PFS, OS, DCR, AEs
NCT05563480	II	Oct 27, 2022	Recruiting	R/M NPC, * N* = 90	TQB2618 + Penpulimab	TIM-3, PD-1	MTD, ORR, PFS	OS, DCR, DOR, AEs, SAEs
NCT03066648	Ib	Jul 6, 2017	Active, not recruiting	AML/HR-MDS, * N* = 241	PDR001 ± MBG453 with HMA	TIM-3, PD-1	AEs, SAEs, DLTs	ORR, DOR, DCR, PFS, TTP, Cmax, Tmax, Half-life
NCT05834543	Ib	May 2023	Not yet recruiting	Advanced ESCC, * N* = 75	TQB2618 + Penpulimab + Chemotherapy	TIM-3, PD-1	PFS, ORR	OS, DCR, DOR, AEs, SAEs
NCT05451407	I	Aug 9, 2022	Not yet recruiting	Advanced Melanoma, * N* = 50	TQB2618 + Toripalimab	TIM-3, PD-1	DLTs, ORR	PFS, OS, DCR
NCT04139902	II	Jun 12, 2020	Recruiting	operable melanoma, * N* = 56	TSR-022 ± TSR-042	TIM-3, PD-1	MPR	PFS, OS, AEs, Frequency of Delays in Surgery
NCT05400876	Ib	Jun 9, 2022	Recruiting	R/R Lymphoma, * N* = 92	TQB2618 + Penpulimab	TIM-3, PD-1	DLTs, ORR	CRR, DCR, DOR, PFS, OS, AEs
NCT03940352	I	Jun 24, 2019	Active, not recruiting	AML, HR-MDS, * N* = 52	MBG453 + HDM201	TIM-3, p53-MDM2	AEs, SAEs, DLTs	ORR, BOR, PFS
NCT05783921	I/II	Mar 2023	Not yet recruiting	R/M-HNSCCs, * N* = 60	TQB2618 + Penpulimab + Chemotherapy	TIM-3, PD-1	PFS, ORR	PFS, OS, DOR, DCR, CBR, AEs, SAEs
NCT03961971	I	Feb 18, 2020	Active, not recruiting	Recurrent GBM, * N* = 16	MBG453 + Spartalizumab + Stereotactic radiosurgery SRS	TIM-3, PD-1	SAEs	grade 3 or higher toxicity, PFS, OS, ORR
NCT05367401	I/II	Dec 20, 2024	Not yet recruiting	Unfit ND-AML/HR-MDS/R/R-AML, * N* = 63	MBG453 + Magrolimab + Azacitidine	TIM-3, CD47	DLTs, CR	ADA, Cmax, Time from first CR to relapse or death, CRR
NCT04370704	I/II	Jul 27, 2020	Recruiting	Melanoma, * N* = 146	INCAGN02385 INCAGN02390 + INCMGA00012	TIM-3, PD-1, LAG-3	TEAEs, ORR, DOR, DCR, PFS	ORR, DCR, PFS
NCT04810611	I	Jun 18, 2021	Recruiting	LR-MDS, * N* = 90	MBG453 ± NIS793 ± Canakinumab	TIM-3, TGF-β, IL-1β	DLTs, AEs, SAEs	BOR, TTP DOR, PFS, ORR
NCT03744468	I/II	Nov 13, 2018	Recruiting	Advanced Solid Tumors, * N* = 358	BGB-A425 + Tislelizumab + LBL-007	TIM-3, PD-1, LAG-3	TEAEs, SAEs, MTD, ORR	DOR, DCR, PFS, PK, Cmax
NCT04586244	II	Jan 14, 2022	Recruiting	Urothelial Carcinoma, * N* = 45	INCAGN02390 + INCAGN02385 + Retifanlimab	TIM-3, LAG-3, PD-1	CD8 + lymphocytes changes	TEAEs, pCR, MPR
NCT05287113	II	Nov 14, 2022	Recruiting	PD-L1-Positive R/M-HNSCCs, * N* = 162	Retifanlimab + INCAGN02385 + INCAGN02390	TIM-3, LAG-3, PD-1	PFS	ORR, DOR, DCR, OS, TEAEs
NCT03307785	I	Oct 12, 2017	Active, not recruiting	Advanced or Metastatic Cancer, * N* = 58	TSR-022 + TSR-042 + Niraparib + Chemotherapy	TIM-3, PD-L1, PARP1/2	DLTs, AEs, TEAEs, STEAEs, AESIs	ORR, DCR, DOR, PFS, ADA, AUC, Cmax
NCT04810611	I	Jun 18, 2021	Recruiting	LR-MDS, * N* = 90	MBG453 ± NIS793 ± Canakinumab	TIM-3, TGF-β, IL-1β	DLTs, AEs, SAEs	BOR, TTP DOR, PFS, ORR

Notes: ADA: Anti-drug Antibody, AEs: Adverse Events, AESIs: Adverse Events of Special Interest, aGvHD: acute Graft-versus-Host Disease, aHSCT: allogeneic Hematopoietic Stem Cell Transplantation, AML: Acute Myelocytic Leukemia, AUC: Area Under the Plasma Concentration Versus Time Curve, BTC: Biliary Tract Carcinomas, BOR: Best Overall Response, CBR: Clinical Benefit Rate, cGvHD: Chronic GVHD, CMML: Chronic Myelomonocytic Leukemia, CR: Complete Response, CRR: Complete Remission Rate, DCR: Disease Control Rate, DLTs: Dose-limiting Toxicities, DOR: Duration of Response, ESCC: Esophageal Squamous Cell Carcinoma, GBM: Glioblastoma Multiforme, HCC: Hepatocellular Carcinoma, irAEs: immune-related Adverse Events, irRC: Immune-related Response Criteria, LFS: Leukemia-free Survival, MPR: Major Pathologic Response, Cmax: Maximum Observed Serum Concentration, MDS: Myelodysplastic Syndromes, MTD: Maximum Tolerated Dose, OR: Objective Response, ORR: Objective Response Rate, OS: Overall Survival, pCR: pathological Complete Response, PK: Pharmacokinetics, PAD: Pharmacologically Active Dose, PFS: Progression-free Survival, RFS: Relapse-free Survival, RO: Receptor Occupation, RP2D: Recommended Phase 2 dose, R/M NPC: Recurrent/Metastatic Nasopharyngeal Carcinoma, R/M-HNSCCs: Recurrent/Metastatic Squamous Cell Carcinoma of the Head and Neck, R/R Lymphoma: Relapsed or Refractory Lymphoma, SAEs: Serious Adverse Events, STEAEs: Serious TEAEs, SD: Stable Disease, SRS: stereotactic radiosurgery, Tmax: Time of Maximum Concentration, TTP: Time to Progress, TEAEs: Treatment-emergent Adverse Events

Clinical studies for three anti-TIM-3 mAbs, including Sym023 (NCT03489343 and NCT03311412), INCAGN02390 (NCT03652077), and sabatolimab (MBG453) (NCT04812548, NCT05020912), have been completed. The safety of these anti-TIM-3 mAbs alone in treating patients with advanced solid tumors or leukemia has been preliminarily demonstrated by the completed studies. In a Phase I/Ib clinical trial for patients with advanced and metastatic solid tumors, sabatolimab treatment alone led to no response; however, five patients who received combination treatment showed partial responses (6%; lasting 12–27 months) [166].

Similarly, different anti-TIM-3 mAbs combined with anti-PD-1 mAbs in the treatment of advanced lymphomas or NSCLC displayed higher efficacy than TIM-3 blockers alone (objective response rate (ORR): 42.9% vs. 0%; disease control rate (DCR): 42.9% vs. 11.1%) [167]. At the ASCO-SITC Clinical Immuno-Oncology Symposium of 2019, it was shown that in a Phase I a/b trial, the efficacy of LY3321367, an anti-TIM-3 mAb, in patients with NSCLC varied depending on the anti-PD-1/L1 efficacy; that is, the efficacy in anti-PD-1/L1 refractory patients (*N* = 23, ORR: 0%, DCR: 35%, PFS: 1.9 months) was compared to that in anti-PD-1/L1 responders (*N* = 14, ORR: 7%, DCR: 50%, PFS: 7.3 months). For patients receiving the combination anti-PD-L1 treatment, the ORR and DCR were 4% and 42%, respectively [168]. In addition, ICIs show higher response rates and durable clinical benefit in microsatellite instability-high/mismatch repair-deficient tumors. A Phase I trial (NCT02791334) demonstrated that combining a TIM-3 inhibitor (LY3321367) with anti-PD-L1 (LY3300054) therapy did not compromise the safety or tolerability of either treatment, and the results suggested numerically higher response rates in the anti–PD-1/PD-L1 inhibitor-naïve microsatellite instability-high/mismatch repair-deficient tumor group (ORR: 45%; DCR: 70%; 1-year OS: 64%, vs. ORR: 33%; DCR: 60%; 1-year OS: 71%) [169]. Clinical trials focused on anti-TIM-3 mAbs combined with inhibitors of the anti-PD-1/PD-L1 axis for the treatment of solid tumors included NCT05400876, NCT04139902, and NCT05645315. Together, the aforementioned data show that although anti-TIM-3 mAbs alone are safe for tumor treatment, the combination treatment blocking the PD-1/PD-L1 axis demonstrates significantly greater effects, and the microsatellite status and ICB treatment history were closely related to efficacy.

In addition, anti-TIM-3 mAbs combined with conventional chemotherapy or demethylation therapy showed increased antitumor effects and are novel options that showed initial efficacy, particularly in the treatment of MDS and AML. The STIMULUS trial (NCT03066648) (data cutoff date June 15, 2021) is another important trial in which 53 patients with very high/high-risk myelodysplastic syndrome (vHR/HR-MDS) and 48 with newly diagnosed AML (ND-AML) were treated with sabatolimab plus a hypomethylating agent (HMA). The incidence of common adverse events (AEs) with a Grade ≥ 3 in both groups was similar to that after HMA administered alone. Higher efficacy was shown for 51 patients with vHR/HR-MDS compared with 40 patients with ND-AML (ORR: 56.9% vs. 40.0%, PFS: 51.9% vs. 27.9%). Moreover, 24.5% of the vHR/HR-MDS patients showed disease attenuation, allowing them to undergo HSCT. Importantly, durable responses were observed in patients with adverse-risk mutations, such as TP53 mutations in vHR/HR-MDS patients (ORR: 71.4%; median duration of response (mDOR): 21.5 months). The AML group presented with a higher mDOR but a higher immune-mediated AE (im-AE) rate than the MDS group (mDOR: 23 vs. 21.5 months, im-AE rate: 25% vs. 11.7%, respectively). Patients with vHR/HR-MDS did not demonstrate excessive GVHD toxicity after subsequent allo-HSCT [96]. Another Phase II clinical study (NCT04150029) reported preliminarily data on the three-drug combination of sabatolimab + venetoclax + azacytidine in 18 ND-AML patients. The addition of 400 and 800 mg sabatolimab led to safety and tolerability comparable to that of the venetoclax + azacytidine combination [170]. A retrospective study of 28 patients with relapsed/refractory (R/R) AML and HR-MDS who received sabatolimab + HMA and subsequently underwent HSCT reported high 2-year PFS (64%) and OS (69%). The data also suggested that treatment with MGB-453 plus MHA before HSCT did not increase posttransplant GVHD and led to improved clinical outcomes for a RAS mutation subgroup, but it did not alter the adverse outcomes of TP53 patients [171]. An additional clinical study (NCT02608268) tentatively demonstrated the safety of TIM-3 inhibitors in the treatment of cancer [96]. In addition to studies focusing on HR-MDS, the program includes a doublet or triplet sabatolimab study for patients with low-risk MDS, which is in the recruiting stage (NCT04810611), for patients with AML that makes them unfit for intensive chemotherapy (STIMULUS-AML1, NCT04150029), and for AML posttransplant patients with measurable residual disease, which is also in the recruiting stage (STIMULUS-AML2, NCT04623216) [172].

In addition, radiotherapy elicits a potent antitumor immune response driven by the activation of T cells infiltrating tumors and an increase in cross-presentation by APCs; these outcomes are considered effective for transforming “cold” tumors into “hot” tumors [173]. Clinical studies analyzing the expression of TIM-3, PD-1, and CTLA-4 on T cells after radiotherapy given to patients with prostate cancer have been conducted to evaluate changes in immune status after radiotherapy (NCT04624828). Similarly, clinical studies on photodynamic therapy are used for basal cell carcinoma (NCT05020912). There are even Phase I clinical trials that use anti-TIM-3 and anti-PD-1 mAbs and stereotactic radiosurgery in combination for the treatment of recurrent glioblastoma multiforme to verify the effectiveness of this strategy (NCT03961971).

### Anti-TIGIT mAbs

Several TIGIT mAbs are being evaluated in clinical trials, with Vibostolimab, Tiragolumab, Ociperlimab, and Domvanalimab being the most advanced candidates in Phase III clinical trials (Table 5).

**Table 5:** Anti-TIGIT mAbs and associated clinical trials in cancer

Clinical trial identifier	Phase	Start date	Status	Cancer type (population, N)	Interventions and Combination	Target	Primary Outcome Measures	Secondary Outcome Measures
NCT04353830	I	May 22, 2020	Complete	Advanced malignancy, * N* = 34	IBI939	TIGIT	AEs, DLTs	AUC, ADA
NCT04354246	I	Mar 31, 2020	Recruiting	Advanced cancer, * N* = 110	COM902	TIGIT	MTD, PK	ORR, CR
NCT04335253	I/IIa	Feb 18, 2020	Completed	Advanced cancer, * N* = 40	EOS-448	TIGIT	RP2D, DLTs	AUC, Cmax
NCT05394168	I	Sep 15, 2022	Not yet recruiting	Advanced/Metastatic Solid Tumor or Lymphoma, * N* = 20	HLX53	TIGIT	MTD, DLTs	ORR, Cmax
NCT03945253	I	Aug 5, 2019	complete	Advanced Solid Tumor, * N* = 6	ASP8374	TIGIT	AEs, DLTs	BOR
NCT04254107	I	May 29, 2020	Recruiting	NSCLC, GC, GEJ, * N* = 417	SAE-TGT	TIGIT	AEs, DLTs	ORR, CR
NCT03563716	II	Aug 10, 2018	Active, not recruiting	NSCLC, * N* = 660	Tiragolumab + Tecentriq	TIGIT PD-L1	ORR, PFS	OS, DOR
NCT04294810	III	Mar 4, 2020	Recruiting	NSCLC, * N* = 136	Tiragolumab + Atezolizumab		OS, PFS	DOR, ORR
NCT05661578	II	May 4, 2023	Recruiting	PD-L1-selected Solid Tumor, * N* = 60	Tiragolumab + Mosunetuzumab or ± atezolizumab	TIGIT PD-L1	AEs	AUC, Cmax
NCT05315713	I/II	May 10, 2022	Active, not recruiting	NHL, FL, * N* = 118	Tiragolumab + Mosunetuzumab or ± Atezolizumab	TIGIT CD20/CD3; TIGIT PD-L1	ORR	CR, DOR
NCT04672369	Ib	Jun 6, 2021	Active, not recruiting	Advanced LCA, * N* = 42	IBI939 + Sintilimab	TIGIT PD-1	ORR	OR, PFS, DCR
NCT04672356	I	Jan 25, 2021	Active, not recruiting	Advanced LCA, * N* = 20			AEs, RP2D	ORR, DCR
NCT02964013	I	Dec 13, 2016	Active, not recruiting	Neoplasms, * N* = 492	Vibostolimab + Pembrolizumab	TIGIT PD-1	DLTs, AEs	ORR, DLTs
NCT05014815	II	Nov 16, 2021	Active, not recruiting	Locally Advanced, Unresectable, or Metastatic NSCLC, NSCLC Stage IV, * N* = 270	Ociperlimab + Tislelizumab + Chemotherapy	TIGIT PD-1	PFS	ORR, DOR, OS
NCT04952597	II	Jul 15, 2021	Active, not recruiting	LS-SCLCr, * N* = 126			PFS	CR, DOR, ORR
NCT04047862	Ib	Aug 26, 2019	Recruiting	Locally Advanced and Metastatic Solid Tumor, * N* = 542	Ociperlimab + Tislelizumab	TIGIT PD-1	DLTs, ORR, SAE	DOR, DCR
NCT05267054	Ib/II	Apr 25, 2022	Recruiting	rrDLBCL, Refractory DLBCL, * N* = 80	Ociperlimab + Tislelizumab/rituximab	TIGIT PD-1/CD20	AEs, RP2D	ORR, DOR
NCT05211895	III	Feb 18, 2022	Recruiting	NSCLC, * N* = 860	Domvanalimab + Durvalumab	TIGIT PD-L1	PFS, BICR	OS, ORR
NCT05130177	II	Mar 16, 2022	Recruiting	Melanoma, * N* = 26	Domvanalimab + Zimberelimab	TIGIT PD-1	ORR, CR, PR	PFS, OS
NCT05568095	III	Nov 21, 2022	Recruiting	Advanced Upper Gastrointestinal Tract Adenocarcinoma, * N* = 970	Domvanalimab + Zimberelimab, Plus Chemotherapy	TIGIT PD-1	OS	PFS, ORR
NCT04826393	I	Mar 9, 2022	Active, not recruiting	GBM, * N* = 14	Domvanalimab + Cemiplimab	TIGIT PD-1	Tmax, MTD	PFS, OS
NCT03260322	I	Sep 8, 2017	Completed	Advanced Solid Tumor, * N* = 169	Domvanalimab + Pembrolizumab	TIGIT PD-1	DLTs, TEAEs	BOR, ORR
NCT05289492	I/II	May 1, 2022	Recruiting	MM, * N* = 162	EOS-448 + iberdomide ± dexamethasone	TIGIT cereblon/GR	SAEs, AEs	PFS, TTR, DCR
NCT05026606	II	Oct 1, 2021	Active, not recruiting	Recurrent-OCCC, Recurrent Platinum-Resistant-FTC, * N* = 20	Etigilimab + Nivolumab	TIGIT PD-1	ORR	irPFS, DCR
NCT04761198	Ib/II	Mar 23, 2021	Active, not recruiting	Solid Tumor Adult, Advanced Solid Tumor, Metastatic Solid Tumor, * N* = 125	Etigilimab + Nivolumab	TIGIT PD-1	ORR	
NCT04570839	I/II	Aug 31, 2020	Active, not recruiting	OV, Solid Tumor, * N* = 100	BMS-986207 + COM701 and Nivolumab	TIGIT PVRIG PD-1	AEs, DLTs	ORR
NCT04585815	Ib/II	Nov 10, 2020	Active, not recruiting	Carcinoma, NSCLC, * N* = 24	BMS-986207 + COM701and Nivolumab	TIGIT PD-1 VEGFR Kit PDGFR	DLTs, CR	DR, TTR
NCT05327530	II	Aug 17, 2022	Recruiting	Locally Advanced or Metastatic UC, *N* = 252	M6223 + Avelumab	TIGIT PD-L1	PFS, TEAEs	OS, OR

ADA: Anti-drug Antibody, AEs: Adverse Events, AUC: Area Under the Plasma Concentration versus Time Curve, BOR: Best Overall Response, BICR: Blinded Independent Central Review, FTC: Carcinoma of Fallopian tube, CR: Complete Response, DLBCL: Diffuse Large B cell Lymphoma, DCR: Disease Control Rate, DLTs: Dose-limiting Toxicities,, DOR: Duration of Response, FL: Follicular Lymphoma, GC: Gastric Cancer, GEJ: Gastroesophageal Junction, GBM: Glioblastoma, iPFS: Immune-related Progression-Free Survival, LS-SCLCr: Limited Stage Small Cell Lung Cancer, LCA: Lung cancer, Cmax: Maximum Observed Serum Concentration, MM: Multiple Myeloma, MTD: Maximum Tolerated Dose, NHL: Non-Hodgkin Lymphoma, NSCLC: Non-small Cell Lung Cancer, OR: Objective Response, ORR: Objective Response Rate, OV: Ovarian Cancer, OCCC: Ovarian Clear Cell Carcinoma, OS: Overall Survival, PK: Pharmacokinetics, PFS: Progression-free Survival, RP2D: Recommended Phase 2 dose, rrDLBCL: Relapsed or Refractory Diffuse Large B cell Lymphoma, SAE: Serious Adverse Event, Tmax: Time of Maximum Concentration, TTR: Time to Response, TEAEs: Treatment-emergent Adverse Events, UC: Urothelial Carcinoma

IBI939 is the first anti-TIGIT mAb approved for clinical trials for leukemia and solid tumor therapy in China [58]. This treatment is a fully human mAb that directly binds to TIGIT to relieve the inhibition and depletion of T cells and NK cells and thus promotes the antitumor effects of these cells. IBI939 is expected to synergistically enhance the antitumor activity of anti-PD-1/PD-L1 mAbs and delay the acquisition of drug resistance. A Phase I clinical trial (NCT04353830) of IBI939 for advanced malignancies established to evaluate its safety, tolerability, pharmacokinetics, and efficacy has been completed.

Vibostolimab (MK-7684) is a humanized immunoglobulin G1 mAb that binds to TIGIT and blocks its interaction with its ligands CD112 and CD155, thereby activating T cells to help kill tumor cells. Vibostolimab plus pembrolizumab was entered into a Phase I clinical trial in 2016 (NCT02964013), and it was well tolerated and showed antitumor activity in patients with advanced solid tumors, including advanced NSCLC [174].

Tiragolumab (RG6058, MTIG7192A) is a fully humanized anti-TIGIT IgG1/kappa mAb developed by Roche with an intact Fc region that blocks the binding of TIGIT to its receptor CD155 [59]. The first Phase I clinical trial of this drug, designed to evaluate tiragolumab alone and in combination with atezolizumab, was conducted in 2016 for patients with advanced/metastatic tumors, including NSCLC and HNSCC (NCT02794571). In this study, 73 patients with multiple tumor types were treated in dose-escalation studies (24 patients were treated with tiragolumab in Phase Ia, and 49 patients were treated with tiragolumab plus atezolizumab in Phase Ib). There was no objective response among the patients in Phase Ia, but stable disease of > 4 months duration was observed (*n* = 4). In the Phase Ib cohort, 3 patients showed responses, with all responses related to PD-L1-positive tumors (2 NSCLC patients: 1 patient with a CR and 1 patient with a PR, and 1 HNSCC patient, who showed a PR), with two patients not receiving prior immunotherapy. Therefore, expansion cohorts were generated for a Phase Ib trial. In the metastatic NSCLC expansion cohort (*N* = 14), the ORR was 50%. Dose-limiting toxicities were not observed in Phase Ia or Ib, and the most common adverse effects were fatigue among the patients in Phase Ia (38%) and anemia among those in Phase Ib (31%) [175]. Tiragolumab combined with other targeted therapy drugs, such as bevacizumab (a vascular endothelial growth factor inhibitor), has also been entered in Phase II clinical trials for patients with NSCLC.

Ociperlimab (BGB-A1217) is a humanized anti-TIGIT IgG1 mAb. This drug binds to the extracellular domain of human TIGIT with high affinity and blocks the interaction between TIGIT and its ligand CD155 or CD112 while preserving intact Fc segment function, which is essential for its antitumor activity; it exerted synergistic antitumor effects when combined with anti-PD-1/PD-L1 mAbs [176]. The results of Phase I clinical trials designed to evaluate ociperlimab combined with tislelizumab (anti-PD-1 mAb) were first reported at ASCO in 2021, and they confirmed that ociperlimab combined with tislelizumab was well tolerated; moreover, antitumor efficacy was observed in patients with advanced solid tumors (NCT04693234) [177]. In June 2023, a Phase III clinical trial evaluating the efficacy and safety of ociperlimab in combination with tislelizumab and platinum-based doublet chemotherapy as first-line treatment for patients with locally advanced or metastatic NSCLC without an operational driver mutation was initiated (NCT05791097).

Domvanalimab (AB154) is another humanized anti-TIGIT mAb that binds human TIGIT and blocks the TIGIT-CD155 interaction, reducing the inhibition of T cells and NK cells, thereby promoting antitumor effects. Moreover, domvanalimab abolished the function of the Fc end of the antibody, which blocked the activity of TIGIT at the nanomolecular level, thereby blocking immunosuppression and increasing immune activity. A Phase III clinical trial evaluating the efficacy and safety of durvalumab (anti-PD-L1 mAb) and domvanalimab compared to that of durvalumab plus placebo in adult patients with locally advanced (Stage III), unresectable NSCLC is currently underway worldwide (NCT05211895) [178].

HLX53, a TIGIT-targeting nano-mAb, is composed of the variable region (VHH) of a heavy chain antibody and the Fc terminus of wild-type IgG1. This drug was approved for a clinical trial of advanced solid tumors or lymphomas in June 2022 by the Center for Drug Evaluation. Preclinical studies have shown that HLX53 exhibited excellent tumor suppression and showed a favorable safety profile [179]. A Phase I clinical trial to evaluate the safety, tolerability, kinetics, and preliminary antitumor efficacy of HLX53 is currently ongoing for patients with advanced/metastatic solid tumors or lymphomas (NCT05394168).

## Other approaches to targeting LAG-3, TIM-3, and TIGIT for immunotherapy

In addition to using mAbs to block ICPs expression and restore tumor-infiltrating immune cell function, several approaches for targeting LAG-3, TIM-3, and TIGIT via immunotherapy, such as the development of BsAbs, have been reported. Since 2009, when the CD3 and CD19 BsAb (a bispecific T cell engager, BiTE) blinatumomab was approved by the FDA for the treatment of Philadelphia chromosome-negative R/R B-ALL [180], increasing numbers of BsAbs have been developed. The clinical therapeutic effects of BsAbs are better than those of mAbs, and BsAbs have a wide range of applications to the treatment of tumors and other diseases [181]. BsAbs that were developed for LAG-3, TIM-3, and TIGIT are summarized in Table 6.

**Table 6:** Clinical trials of anti-LAG-3, anti-TIM-3, anti-TIGIT BsAbs

Clinical trial identifier	Phase	Start date	Status	Cancer type (population, *N*)	Interventions and combination	Target	Primary outcome measures	Secondary outcome measures
NCT03219268	I	Aug 18, 2017	Completed	Unresectable or Metastatic Neoplasms, *N* = 353	Tebotelimab (MGD013)	LAG-3 PD-1	TEAEs, etc	AUC, Cmax, etc
NCT04082364	II/III	Sep 30, 2019	Active, not recruiting	GC, GEJC, HER2 + GC, *N* = 82			ORR, CR, PR, etc	ADA, etc
NCT04634825	II	Mar 17, 2021	Terminated	HNC, HNSCC, *N* = 62			ORR, CR, etc	BOR, Cmax, etc
NCT05419388	I/II	Aug 15, 2022	Recruiting	Melanoma, *N* = 80	RO7247669	LAG-3 PD-1	PFS	ORR, DOR, etc
NCT05645692	II	Apr 13, 2023	Recruiting	UC, *N* = 240			ORR	OS, DCR, etc
NCT04140500	I/II	Nov 11, 2019	Recruiting	Solid tumor, NSCLC, metastatic melanoma, *N* = 320			DLTs, ORR, etc	Cmax, AUC, etc
NCT04785820	II	Jun 25, 2021	Recruiting	AMESCC, *N* = 210			OS	ORR, DCR, etc
NCT05805501	II	Apr 21, 2023	Recruiting	RCC, *N* = 210			PFS	OS, ORR, etc
NCT05775289	II	Mar 15, 2023	Recruiting	NSCLC, *N* = 180			PFS, ORR	OS, DOR, etc
NCT04524871	I/II	Nov 2, 2020	Recruiting	Advanced LC, *N* = 400			ORR	PFS, OS, etc
NCT03440437	I/II	Apr 16, 2018	Recruiting	AMC, HNSCC, *N* = 80	FS118	LAG-3 PD-L1	AUC, CL, etc	Cmax, Tmax, etc
NCT04618393	I/II	Mar 11, 2021	Recruiting	Advanced solid tumors, *N* = 43	EMB-02	LAG-3 PD-1	SAEs, ORR, etc	AUC, Cmax, etc
NCT04916119	I	Jun 29, 2021	Recruiting	Advanced malignancies, *N* = 322	IBI323	LAG-3 PD-L1	AEs, etc	ORR, DCR, etc
NCT03849469	I	May 29, 2019	Completed	Melanoma, CESC, PAAD, TNBC, HCC, BLCA, etc, *N* = 78	XmAb22841	LAG-3 CTLA-4	AEs	–
NCT05695898	I/II	Feb 28, 2023	Recruiting	AM Melanoma, *N* = 46			AEs, DLTs, etc	AUC, Cmin, etc
NCT03752177	Ia/Ib	Nov 22, 2018	Terminated	Advanced Solid Tumors, *N* = 12	LY3415244	TIM-3 PD-L1	DLTs	ORR, DOR, etc
NCT03708328	I	Oct 15, 2018	Active, not recruiting	Advanced and/or metastatic solid tumors, *N* = 134	Lomvastomig (R07121661)	TIM-3 PD-1	DLTs, etc	AUC, Cmax, etc
NCT04785820	II	Jun 25, 2021	Recruiting	AMESCC, *N* = 210	Lomvastomig (R07121661)	TIM-3 PD-1	OS	ORR, DOR, etc
NCT04931654	I/IIa	Sep 28, 2021	Recruiting	NSCLC, other AST, *N* = 81	AZD7789	TIM-3 PD-1	AEs, DLTs, etc	OS, etc
NCT05216835	I/II	Mar 18, 2022	Recruiting	R/R HL, *N* = 180	AZD7789	TIM-3 PD-1	AEs, DLTs, etc	CRR, ORR, etc
NCT05357651	I	Aug 12, 2022	Recruiting	Advanced solid tumors or lymphoma, *N* = 100	LB1410	TIM-3 PD-1	TEAEs, SAEs, etc	ORR, DCR, etc
NCT05005442	II	Sep 28, 2021	Recruiting	HMs, *N* = 180	MK-7684A	TIGIT PD-1	DLTs, AEs	ORR, DOR, etc
NCT04911881	Ia	Jun 24, 2021	Completed	Advanced solid tumors, *N* = 36	IBI321	TIGIT PD-1	DLTs, TRAE	ORR, PFS, etc
NCT04911894	I	Jun 21, 2021	Completed	Advanced solid tumors, *N* = 16			AEs, DLTs	DOR, PFS, etc
NCT04995523	I/II	Sep 14, 2021	Recruiting	NSCLC, *N* = 192	AZD2936	TIGIT PD-1	AEs, DLTs, ORR	DCR, DOR, etc
CTR20220021	II	Nov 5, 2021	Recruiting	Advanced solid tumors, *N* = 30	ZG005	TIGIT PD-1	DLTs, AEs	DCR, DOR, etc
NCT05025085	I	Oct 4,2021	Active, not recruiting	Advanced cancer, *N* = 70	AGEN1777	TIGIT an undisclosed target	DLTs, TEAEs	ADA, CR, etc

*ADA* antidrug antibody, *AEs* adverse events, *AM* advanced and/or metastatic, *AMC* advanced and/or metastatic cancer, *AMESCC* advanced and/or metastatic esophageal squamous cell carcinoma, *AUC* area under the plasma concentration versus time curve, *BOR* best overall response, *BLCA* bladder cancer, *CESC* cervical and endocervical cancers, *CL* clearance, *CR* complete response, *CRR* complete remission rate, *DCR* disease control rate, *DLTs* dose-limiting toxicities, *DOR* duration of response, *GC* gastric cancer, *GEJC* gastroesophageal junction cancer, *HCC* hepatocellular carcinoma, *HNC* head and neck cancer, *HNSCC* head and neck squamous cell carcinoma, *HMs* hematological malignancies, *LC* liver cancers, *Cmax* maximum observed serum concentration, *Cmin* minimum serum concentration, *NSCLC* non-small cell lung cancer, *ORR* objective response rate, *OS* overall survival, *PAAD* pancreatic adenocarcinoma, *PR* partial response, *PFS* progression-free survival, *R/R HL* relapsed/refractory Hodgkin lymphoma, *RCC* renal cell carcinoma, *SAEs* serious adverse events, *Tmax* time of maximum concentration, n, *TEAEs* treatment-emergent adverse events, *TRAE* treatment-related AE, *TNBC* triple-negative breast cancer, *UM* unresectable or metastatic, *UC* urothelial cancer

### LAG-3-PD-1/PD-L1/CTLA-4/TIGIT BsAbs

Four types of BsAbs have been developed for LAG-3: LAG-3-PD-1, LAG-3-PD-L1, LAG-3-CTLA-4, and LAG-3-TIGIT. The first LAG-3-PD-1 BsAb developed was tebotelimab (MGD013), which specifically binds to PD-1 and LAG-3 with high affinity and targets cell lines expressing these proteins and chronically activated T cells. Functional characterization of tebotelimab revealed enhanced cytokine secretion in response to antigen rechallenge of previously stimulated T cells treated with tebotelimab compared to that after PD-1 or LAG-3 pathway blockade alone. In a Phase I clinical trial for relapsed or refractory DLBCL patients, after tebotelimab treatment, serum IFN-γ levels were found to be significantly elevated, and CD8^+^ T cell function was restored with an increase in cytolytic marker (i.e., perforin, granzyme B) levels. Encouraging early evidence suggests that tebotelimab exhibits good pharmacodynamic, safety and antitumor activity, regardless of whether patients with R/R DLBCL had previously received chimeric antigen receptor T cell (CAR-T) therapy [182]. Tebotelimab was used in combination with margetuximab, a HER2^−^targeting mAb, for patients with HER2^+^ BC therapy [183]. There are currently seven clinical trials evaluating tebotelimab monotherapy or combination therapy. In addition, tebotelimab monotherapy has shown antitumor activity in multiple tumor types, such as melanoma and advanced HCC [184]. There is a newly launched LAG-3-PD-1 BsAb, CB213, and its structure is composed of a human nanobody (recombinant variable domains of a heavy-chain-only antibody) with an asymmetric 2:1 binding format involving bivalent human LAG-3 and monovalent human PD-1. The antitumor efficacy of CB213 was characterized by the potent inhibition of tumor growth and an increase in the number of CD8^+^ T cells with tumor antigen specificity [185]. Another LAG-3-PD-1 BsAb, RO7247669, targets and binds PD-1/LAG-3^+^ T cells and leads to CTL-induced immune responses against tumor cells [186]. The safety and efficacy of RO7247669 are still being evaluated in the contexts of metastatic melanoma, NSCLC, esophageal squamous cell carcinoma (ESCC), and advanced HCC. Additionally, EMB-02 is a LAG-3-PD-1 BsAb that has been shown to restore effector T cell function and enhance antitumor activity (NCT04618393).

Two LAG-3-PD-L1 BsAbs have been developed, FS118 and IBI323. FS118 targets LAG-3 and PD-L1 and shows the potential to activate exhausted immune cells and to target and overcome resistance to PD-L1 blockade. FS118 binds both LAG-3 and PD-L1, blocking PD-1/PD-L1, CD80/PD-L1, and LAG-3/MHC-II interactions, thereby reversing T cell suppression and promoting the production of cytokines by CD4^+^ and CD8^+^ T cells [187]. A Phase I clinical trial (NCT03440437) for patients with advanced cancers and PD-L1 drug resistance initially showed that FS118 was well tolerated, but further studies are needed to determine the clinical benefit for patients refractory to anti-PD-(L)1 therapy [188]. IBI323 demonstrated similar potency in blocking the interactions of PD-1/PD-L1, CD80/PD-L1, and LAG-3/MHC-II. In PD-L1/LAG-3 double knock-in mice bearing human PD-L1 knock-in MC3 tumors, IBI323 exhibited stronger antitumor activity than each parental antibody, and these antitumor responses were associated with an increase in the number of tumor-specific CD8^+^ and CD4^+^ T cells [189].

XmAb22841 is a CTLA-4-LAG-3 BsAb developed by Xencor that enhances T cell activation. The structure of XmAb22841 consists of a bispecific Fc domain that functions as a scaffold between the two binding domains, conferring stability and making purification and fabrication easy. This structure promotes heterodimer formation and leads to a long half-life in the circulatory system. XmAb22841 enhances allogeneic antitumor activity and facilitates triple checkpoint blockade in combination with anti-PD-1 blockade [190]. There are two ongoing clinical trials for evaluating XmAb22841 as a melanoma treatment, either alone or in combination with pembrolizumab or XmAb23104 (PD-1 × ICOS) (NCT03849469, NCT05695898).

ZGGS15 is a humanized anti-LAG-3 and anti-TIGIT BsAb that not only blocks the signaling pathways activated by LAG-3 and its ligand MHC-II but also activates the TCR signaling pathway, thereby promoting the activation and proliferation of T and NK cells and cytokine production, synergistically enhancing the ability of the immune system to kill tumor cells. The combination of ZGGS15 and anti-PD-1 mAb showed high efficacy than anti-LAG-3 mAbs or anti-TIGIT mAbs combined with anti-PD-1 mAbs [190]. In June 2023, the FDA approved ZGGS15 injection for the treatment of patients with advanced solid tumors in a clinical trial (NCT05864573).

### TIM-3-PD-1/PD-L1 BsAbs

In preclinical studies, a PD-1-TIM-3 BsAb was shown to increase the abundance of activated (HLA-DR^+^CD25^+^GranzymeB^+^) and proliferating CD8^+^ T cells and NK cells [191].

Compared with anti-PD-1 antibodies alone, TIM-3-PD-1 BsAbs significantly increased the proportion of proliferating NK cells [191]. In addition, 45% (5/11) of samples from patients with solid tumors demonstrated an effective response, namely a twofold in IFN-γ secretion after 96 h of coculture with PD-1-TIM-3 BsAbs in vitro [191]. Moreover, in responsive tumor samples, TIM-3-PD-1 BsAbs indirectly promoted B cell activation by inhibiting the production of PD-1^+^CXCL13^+^CD4^+^ T cells [191]. Mechanistically, the chemokine CXCL13 in the TME may play a crucial role as a B cell attractant [192]. Another PD-1-TIM-3 BsAb, MCLA-134, is currently being evaluated in preclinical studies [193].

To date, two types of BsAbs, TIM-3-PD-1 and TIM-3-PD-L1, have been entered into clinical trials. The TIM-3-PD-L1 BsAb, known as LY3415244, was entered into a Phase Ia/Ib study for the treatment of advanced solid tumors in November 2018, and it was terminated early in October 2019 due to an unpredictable immune response (NCT03752177). Two patients (16.7%) developed clinically significant infusion-related anaphylactic reactions, and all 12 patients developed treatment-related antidrug antibodies [194]. Although the study was stopped early, one patient with NSCLC who was resistant to PD-1 blockade achieved a near partial response with tumor regression by 29.6% [194].

The first TIM-3-PD-1 BsAb, R07121661, was used in a Phase I study to treat advanced and/or metastatic solid tumors, including ESCC, melanoma, SCLC, and NSCLC (NCT03708328), and in a Phase II study, it was used to treat advanced or metastatic ESCC (NCT04785820) [195]. The second TIM-3-PD-1 BsAb, AZD7789, was used in a Phase I/II open-label, multicenter study designed to assess its safety, tolerability, pharmacokinetics, and preliminary efficacy in patients with R/R classical HL (NCT05216835) and advanced solid tumors (NCT04931654). These studies are currently in the recruitment stage. Based on adjustments to both the heavy and light chain complementarity-determining regions, differences in the TIM-3-PD-1 BsAbs are described in detail in patents EP33564A1 and WO2019009727 [196].

### TIGIT-PD-1/CTLA-4 BsAbs

A few clinical trials have shown that anti-TIGIT mAbs administered in combination with various other ICIs are much more effective than monotherapy, but determination of the optimal combinations with different ICIs remains a challenge. Therefore, many dual-target antibodies targeting TIGIT and other ICPs have been developed. BsAbs compensate for a deficiency of single drug therapy and generally show greater safety and efficacy.

IBI321 was the first dual-targeting IC (TIGIT and PD-1) BsAb to enter clinical practice. This molecule inhibits both the PD-1 and TIGIT signaling pathways. On July 26, 2021, Cinda announced completion of the first Phase I clinical trial of IBI321 with Chinese patients. The study was established mainly to evaluate the safety, tolerability, and antitumor activity of the dual-target antibody IBI321 in 16 patients with advanced malignant solid tumors who failed to respond to standard treatment (NCT04911894). The trial was completed on February 17, 2023, but no data on the drug have been reported.

The second PD-1-TIGIT BsAb is ZG005, which was declared for clinical application in China and is mainly suitable for advanced malignant tumors. As a dual blockade of the PD-1/TIGIT ICPs, ZG005 showed sustained occupancy of both targets and was able to specifically inhibit their pathways simultaneously, resulting in synergistic effects and enhanced ability of the immune system to kill tumor cells. A Phase I clinical trial (CTR20220021) of ZG005 in advanced cancer is ongoing [197].

Another PD-1-TIGIT BsAb treatment is BC008-1A injection. This BsAb can enhance the role of immune surveillance by recognizing and killing tumor cells and blocking the potential synergistic effects of PD-1 and TIGIT to enhance antitumor effects. This treatment is suitable mainly for patients with advanced solid tumors and was approved for clinical trials in September 2022. A Phase I clinical trial (CTR20230047) is still in the recruitment phase, and 36 patients are expected to be enrolled. The aim of this trial is primarily to evaluate the safety and tolerability of BC008-1A injection in subjects with advanced solid tumors and determine the dose-limiting toxicities and maximum tolerated dose.

In addition, Yang et al. developed an anti-TIGIT mAb that exerts strong antitumor effects through mechanisms including a CD8^+^ T immune response and Fc-mediated effector functions that cause a significant reduction in the number of intratumoral Tregs. This result suggests that TIGIT-Fc treatment alone or in combination with other checkpoint receptor blockers is a promising anticancer therapeutic strategy [198]. In 2021, Bristol Myers Squibb and Agenus Inc. announced a license for a BsAb, AGEN1777, which blocks TIGIT and a second undisclosed target. The enhanced Fc region shows higher binding affinity for T and NK cells, thus increasing the activation of these cells [199]. This BsAb is being evaluated to determine its safety, tolerability, pharmacokinetics, and pharmacodynamics as a single agent and in combination with PD-1 inhibitors in patients with advanced solid tumors (NCT05025085).

In contrast to dual ICP BsAbs, AK130 is a TIGIT/human transforming growth factor-β (TGF-β) dual target antibody fusion protein independently developed by Kangfang Biological. This BsAb is composed of an anti-TIGIT mAb fused to the extracellular domain of TGF-β receptor II, and it is the first and only TIGIT/TGFβ dual target fusion protein antibody developed to date. In preclinical studies, AK130 blocked the TIGIT-CD155 and TGFβ-TGFβR signaling pathways, and it showed a profound ability to increase IL-2 secretion. Moreover, AK130 exhibited significant antitumor activity without inducing antibody-dependent cell-mediated cytotoxicity or complement-dependent cytotoxic effects in HCC model mice. Overall, AK130 simultaneously targets TIGIT and TGF-β to relieve immunosuppression, activate antitumor immune responses, and inhibit tumors at the same time [200]. It will be evaluated for its safety, tolerability, pharmacokinetics, and antitumor activity in patients with advanced malignancies in a Phase I trial (NCT05653284). Recruitment has not yet begun, and the trial is expected to be completed in 2025.

### sLAG-3-Ig fusion protein induces APC activation

Eftilagimod alpha (IMP321), developed by IMMUTEP S.A.S., is an original, first-in-class LAG-3 regulator, and it is the only soluble recombinant LAG-3 used in clinical research. Eftilagimod alpha is a fusion protein consisting of the LAG-3 extracellular domains fused to a human Ig Fc region, and it was developed by replacing the Fab Ig domains of IgG1 with four Ig-like domains from the extracellular region of LAG-3. Eftilagimod alpha activates APCs through its interaction with MHC-II. Eftilagimod alpha interaction with MHC-II on human immature DCs induces the secretion of IL-12 and TNF-α, and it promotes morphological changes, such as the formation of dendritic projections. Hence, eftilagimod alpha functions differently from antagonistic LAG-3 mAbs that block the LAG-3/MHC-II interaction and thus the LAG-3-mediated reducing in T cells [201, 202]. Five clinical trials of eftilagimod alpha have been completed (NCT02676869, NCT02614833, NCT00349934, NCT00351949, and NCT00324623), and other trials are ongoing. The design of most of these trials is based on combination treatments. Combining eftilagimod alpha1 and PD-1 mAbs for treating patients with metastatic melanoma demonstrated that eftilagimod alpha was well tolerated and showed encouraging antitumor activity. An ORR of 33% was observed in patients refractory to pembrolizumab treatment in the dose-escalation part of the study, and an ORR of 50% was observed for PD-1-naïve patients in the extension part of the study [203].

### Small molecules targeting TIM-3

The small-molecule LPX-TI641 is an orally bioavailable TIM family agonist (TIM-3 and TIM-4) designed to promote immune tolerance restoration/induction. As an orally administered therapeutic, LPX-TI641 represents a new approach that unleashes the power of immune tolerance while being unrestrained by the limitations of previous antigen-specific immune tolerance approaches. LAPIX Therapeutics initially designed immune system restoration therapies for both autoimmune disease and oncology applications, but only LPX-TI641 has been entered into a Phase I clinical trial, and its safety and efficacy in neuro-autoimmune indications such as multiple sclerosis are being evaluated (NCT05853835).

## Combination of targeting LAG-3, TIM-3, and TIGIT mAbs with other immune therapy strategies

CAR-T cell therapy has been widely used to treat B cell malignancies [204]. Current challenges to the use of CAR-T cell therapy include failure to persist and produce prolong antitumor responses in the immunosuppressive TME [204, 205]. The response rate of CAR-T cell therapy for B cell malignancies is different. To understand why only 26% of CLL patients benefited from CD19 CAR-T therapy while more than 90% of CD19^+^ B-ALL patients experienced CR, a detailed transcriptomic analysis was performed to compare CLL responses after CD19 CAR-T therapy using CAR-T cells from responders and non-responders. CAR-T cells from non-responders upregulated pathways involved in exhaustion and apoptosis [206, 207]. The expression levels of ICPs such as PD-1, TIM-3, and LAG-3 were upregulated on CAR-T cells after infusion, which may be related to CAR-T cell dysfunction [208, 209]. Moreover, CAR-T cells deficient in PD-1 or LAG-3 demonstrated better antitumor efficacy both in vitro and in vivo [210]. In mesothelin-CAR-T cells, a lower expression level of the exhausted phenotype, including PD-1, LAG-3, and TIM-3 expression, has been shown to increase the strength and prolong clinical responses in the treatment of OC models [211].

Significantly, T cell exhaustion induced by coinhibitory pathways has been thought to contribute to the low persistence and highly dysfunctional activity of CAR-T cells. Thus, several studies have explored selective blockers of these inhibitory receptors in CAR-T cells. To date, at least two TIM-3-CD28 fusion proteins have been designed to increase the proliferation, activation, and cytotoxic capacity of conventional anti-CD19 CAR-T cells [212]. In addition, TIGIT has been identified is a marker for CD19 CAR-T cell dysfunction in experiments involving single-cell RNA sequencing and in analysis of surface protein marker levels before and after CAR-T cell infusion in NHL patients. Simultaneous downregulation of PD-1 and TIGIT enhances the in vivo function of CD19 CAR-T cells, resulting in coordinated antitumor effects [213]. TIGIT inhibition alone has also been shown to increase the efficacy of CAR-T cells [214]. Moreover, evidence suggests that TIGIT is highly expressed in mantle cell lymphoma cells in patients after relapse, and cotargeting TIGIT prevented CAR-T cell relapses, thereby promoting the long-term PFS of mantle cell lymphoma patients [215]. In addition, a group of investigators constructed anti-MLSN-CAR-T cells combined with anti-α-TIGIT for the treatment of solid tumors, e.g., pancreatic cancer, BC, and OC. Blocking TIGIT significantly promoted the release of cytokines, thereby enhancing the tumor-killing effects of the anti-MLSN-CAR-T cells. Moreover, anti-α-TIGIT scFv expression and secretion interrupted the interaction between TIGIT and its ligand CD155, enhancing the infiltration and activation of CAR-T cells in the TME to achieve increased tumor regression in vivo [216]. Additionally, TIM-3 was used to develop a second-generation 41BB-CD19-CAR linked with a switch receptor T3/28 chimera, known as T3/28 CAR-T cells, which significantly prolonged the persistence of CAR-T cells and showed potent antitumor activity both in vitro and in MM model mice [217]. Moreover, TIM-3 is expressed on most LSCs of AML but not on normal HSCs [41, 106]. The first anti-TIM-3 CAR-T cell was designed and demonstrated effective anti-myeloid leukemia effects both in vitro and in AML model mice [218].

Similar to CAR-T cells, BiTEs target tumor-specific antigens but depend on the normal function of T cells [219]. In the multicohort, open-label, phase 1/2 MajesTEC-1 study, in which the safety/efficacy of teclistamab (a B cell maturation antigen (BCMA)-CD3 BsAb IgG4) were evaluated in patients with RR-MM, encouraging efficacy was demonstrated and indicated that a higher frequency of T cells expressed IC markers, including TIM-3, and this may be an underlying reason for non-responders observed with unfavorable immune characteristics at baseline [220]. Thus, a combination of BiTEs with ICIs may be increase the effect of the BiTEs.

## Conclusion and perspectives

LAG-3, TIM-3, and TIGIT are the next wave of targets for ICBs, and their efficacy has been extensively evaluated in clinical trials, following the evaluation of PD-1/PD-L1 and CTLA-4 blockers [1, 10]. In addition to single-target blockade, dual inhibition of PD-1/PD-L1 and other ICPs, such as CTLA-4, LAG-3, TIM-3, and TIGIT, has been tested in patients with different types of cancer in recent years. Encouragingly, the FDA approved the first dual inhibitor of LAG-3 and PD-1 drug (Opdualag) for treating adult and pediatric patients with unresectable or metastatic melanoma [7]. In addition to the combined application of two ICIs, BsAbs targeting two ICPs is a strategy to overcome cell resistance to a single ICB [221, 222]. This year, the FDA approved injection of an anti-LAG-3/TIGIT BsAb for the treatment of patients with advanced solid tumors in clinical trials. However, whether the BsAbs targeting two ICPs exhibit a “1 + 1 > 2” effect compared with the combined use of two types of ICP mAbs and induce fewer side effects remains to be seen in the future. Moreover, it is known that ICP-mediated exhaustion significantly influences the function of CAR-T cells, and ICB can increase CAR-T cell function. However, the determination of which ICI is the best choice for reversing CAR-T cell function requires further investigation. Finally, ICBs combined with CAR-T cells, BiTEs, and tumor vaccines and the development of BsAbs targeting one ICP and one immune suppressive cytokine are promising strategies to overcome immune escape by different types of cancer cells. The development of these new ICP-based treatment strategies offers new hope for cancer patients; however, to develop a strategy for choosing combination therapy targets for specific types of tumors and specific individuals, further exploration is needed. The identification of the most efficacious combination might depend on additional exploration into ICP expression profiles in different types of tumors and a deeper understanding of the molecular mechanisms underlying the effect of each ICP. Undoubtably, only a set of biomarkers that can predict the efficacy of ICBs will enable guided clinical drug administration.

## Data Availability

Data sharing is not applicable to this article as no datasets were generated or analyzed during the current study.

## Abbreviations

AE: Adverse events
AML: Acute myeloid leukemia
APCs: Antigen-presenting cells
BC: Breast cancer
B-ALL: B cell acute lymphoblastic lymphoma
BsAbs: Bispecific antibodies
BiTE: Bispecific T cell engager
CEACAM-1: Carcinoembryonic antigen cell adhesion molecule 1
CC: Colon cancer
CR: Complete response
CTL: Cytotoxic T lymphocyte
CTLA-4: Cytotoxic T lymphocyte antigen-4
CLL: Chronic lymphocytic leukemia
CAR-T cell: Chimeric antigen receptor T cell
DCR: Disease control rate
DCs: Dendritic cells
DLBCL: Diffuse large B cell lymphoma
ESCC: Esophageal squamous cell carcinoma
FGL-1: Fibrinogen-like protein 1
FDA: Food and Drug Administration
Gal-9: Galectin-9
GC: Gastric cancer
GVHD: Graft-versus-host disease
IFN: Interferon
ICPs: Immune checkpoint proteins
ICB: Immune checkpoint blocker
ICI: Immune checkpoint inhibitor
IL: Interleukin
ITIM: Immunoreceptor tyrosine-based inhibitory motif
LAG-3: Lymphocyte activation gene-3
LAP: LAG-3-related protein
LSECtin: Liver sinusoidal endothelial cell lectin
LSCs: Leukemia stem cells
mAb: Monoclonal antibody
MHC-II: MHC class II
MDS: Myelodysplastic syndromes
mDOR: Median duration of response
mDCs: Monocyte-derived DCs
ND-AML: Newly diagnosed AML
HCC: Hepatocellular carcinoma
HL: Hodgkin lymphoma
HMGB1: High-mobility group protein B1
HNSCC: Head and neck squamous cell carcinoma
NHL: Non-Hodgkin lymphoma
HR: High risk
NK: Natural killer cells
HMA: Hypomethylating agent
NSCLC: Non-small cell lung cancer
ORR: Objective response rate
OS: Overall survival
OC: Ovarian cancer
allo-HSCT: Allogeneic hematopoietic stem cell transplantation
PD-1: Programmed cell death protein 1
PFS: Progression-free survival
PR: Partial response
PtdSer: Phosphatidylserine
RCC: Renal cell carcinoma
RIG-I: Retinoic acid-inducible gene I
R/R: Relapsed/refractory
sTIM-3: Soluble form of TIM-3
TME: Tumor microenvironment
TCR: T cell receptor
TIGIT: T cell immunoreceptor with immunoglobulin and ITIM domain
TIM-3: T cell immunoglobulin and mucin-domain-containing-3
Tregs: Regulatory T cells
TILs: Tumor-infiltrating lymphocytes
TGF-β: Transforming growth factor-β
SCLC: Small cell lung cancer
vHR: Very high risk
