# Correction: Targeting LAG-3, TIM-3, and TIGIT for cancer immunotherapy

**DOI:** 10.1186/s13045-023-01503-8

**Published:** 2023-09-29

**Authors:** Letong Cai, Yuchen Li, Jiaxiong Tan, Ling Xu, Yangqiu Li

**Affiliations:** 1 Key Laboratory for Regenerative Medicine of Ministry of Education, Institute of Hematology, School of Medicine, Jinan University, Guangzhou, 510632 China; 2 Key Laboratory of Viral Pathogenesis & Infection Prevention and Control (Jinan University), Ministry of Education, Guangzhou, 510632 China

**Correction to: Cai et al. Journal of Hematology & Oncology (2023) 16:101** 10.1186/s13045-023-01499-1

The original article [1] contained an error mistakenly introduced by the production team whereby co-first authorship for the first three co-authors was omitted. This has since been restored accordingly.
